# RanBP2/Nup358 Potentiates the Translation of a Subset of mRNAs Encoding Secretory Proteins

**DOI:** 10.1371/journal.pbio.1001545

**Published:** 2013-04-23

**Authors:** Kohila Mahadevan, Hui Zhang, Abdalla Akef, Xianying A. Cui, Serge Gueroussov, Can Cenik, Frederick P. Roth, Alexander F. Palazzo

**Affiliations:** 1 Department of Biochemistry, University of Toronto, Toronto, Ontario, Canada; 2 Department of Biological Chemistry & Molecular Pharmacology, Harvard Medical School, Boston, Massachusetts, United States of America; 3 Howard Hughes Medical Institute, Department of Biochemistry and Molecular Pharmacology University of Massachusetts Medical School, Worcester, Massachusetts, United States of America; 4 Donnelly Centre for Cellular and Biomolecular Research, University of Toronto, Toronto, Ontario, Canada; 5 Samuel Lunenfeld Research Institute, Mt. Sinai Hospital, Toronto, Ontario, Canada; Scripps Research Institute, United States of America

## Abstract

In higher eukaryotes, most mRNAs that encode secreted or membrane-bound proteins contain elements that promote an alternative mRNA nuclear export (ALREX) pathway. Here we report that ALREX-promoting elements also potentiate translation in the presence of upstream nuclear factors. These RNA elements interact directly with, and likely co-evolved with, the zinc finger repeats of RanBP2/Nup358, which is present on the cytoplasmic face of the nuclear pore. Finally we show that RanBP2/Nup358 is not only required for the stimulation of translation by ALREX-promoting elements, but is also required for the efficient global synthesis of proteins targeted to the endoplasmic reticulum (ER) and likely the mitochondria. Thus upon the completion of export, mRNAs containing ALREX-elements likely interact with RanBP2/Nup358, and this step is required for the efficient translation of these mRNAs in the cytoplasm. ALREX-elements thus act as nucleotide platforms to coordinate various steps of post-transcriptional regulation for the majority of mRNAs that encode secreted proteins.

## Abstract 2

After nuclear export, mRNAs encoding secretory proteins interact with RanBP2/Nup358 on the cytoplasmic face of the nuclear pore, a step that is required for the efficient translation of these mRNAs.

## Author Summary

About one-fifth of the protein-coding genes in the human genome code for secreted and/or membrane-bound proteins. In the nucleus these genes are transcribed into messenger RNAs (mRNAs), which are then exported to the cytoplasm. These mRNAs are then transported to the surface of the endoplasmic reticulum where they are translated into proteins destined for the secretory pathway. Most of these mRNAs contain signal sequence coding regions (SSCRs), which code for short hydrophobic polypeptides that target the newly synthesized proteins for translocation across the endoplasmic reticulum membrane. Previously, we found that many SSCRs also act as RNA elements that promote the efficient nuclear export of mRNAs. Here we present evidence that upon the completion of nuclear export, SSCR-containing mRNAs interact with RanBP2/Nup358, a large protein found on the cytoplasmic face of the nuclear pore. This interaction is mediated by direct binding between the SSCR and zinc finger repeats found within RanBP2/Nup358, and is ultimately required for the efficient translation of SSCR-containing mRNAs into secretory and/or membrane-bound proteins. Our work demonstrates that SSCRs act as nucleotide platforms that recruit various factors to the mRNA throughout its life to regulate distinct events, such as nuclear export and translation.

## Introduction

In eukaryotic cells, mRNA synthesis and processing occur in the nucleus while the translation of mRNA into protein is restricted to the cytoplasm. Although these various mRNA metabolic steps take place in distinct compartments, these events are biochemically coupled [1],[2]. For example, the 5′ cap binding complex and the spliceosome collaborate to deposit the transcription export (TREX) complex on the 5′ end of a newly synthesized transcript [3]. TREX then recruits the TAP/p15 heterodimer, which ultimately allows the mRNA to be exported from the nucleus into the cytoplasm [4],[5].

Despite the fact that the vast majority of transcripts contain introns and should therefore use the splicing-dependent export mechanism, we previously demonstrated that most mRNAs that encode secreted proteins contain RNA elements that promote an alternative mRNA nuclear export (ALREX) pathway that is independent of both splicing and a 5′ cap structure [6]. In addition, these ALREX-promoting sequences are found within the signal sequence coding region (SSCR) at the 5′ end of the ORF. SSCRs from vertebrates (and to a lesser extent in invertebrates) tend to contain long stretches of nucleotide sequence that lack adenine. This depletion in adenines is due to the enrichment in both amino acids that are encoded by adenine-poor codons and synonymous codons lacking adenine. Indeed, ALREX activity can be inhibited when nucleotides within the SSCR are silently substituted for adenines, so that the encoded amino acid remains unaltered [6].

Recently, we demonstrated that SSCR-containing genes tend to lack introns in their 5′ UTR (i.e., upstream of the SSCR) [7]. When SSCRs were present in genes that contained 5′ UTR introns, these SSCRs were not as depleted of adenines, and did not promote the export of a reporter mRNA [7]. These results suggested that the 5′ most element in a transcript, be it an intron or SSCR, dictates whether the mRNA is exported by either the splicing or ALREX pathway. This model is supported by the observation that ALREX-promoting elements only potentiate export when present near the 5′ end of a reporter transcript [8].

Interestingly, the incorporation of silent adenine mutations into the ALREX-promoting SSCR not only inhibited nuclear export, but also induced the formation of cytoplasmic stress granules (SGs) into which the mutated mRNAs partially accumulated [6]. Typically, these cellular structures form in response to an accumulation of cytoplasmic transcripts that fail to initiate proper translation [9],[10]. This observation suggested that the ALREX-promoting element might influence not only export, but also other downstream events, such as how the mRNA is localized in the cytoplasm and how efficiently the translation-initiation complex is assembled onto the transcript.

Here we provide evidence that ALREX-promoting elements potentiate translation. Furthermore, our data indicate that these RNA elements associate with the zinc finger repeats (ZFRs) of RanBP2, a large protein found on the cytoplasmic face of the nuclear pore complex. We then demonstrate that the depletion of RanPB2 not only prevents the normal potentiation of translation by ALREX-promoting sequences, but also inhibits the global synthesis of secreted and likely mitochondrial proteins.

## Results

### ALREX-Promoting SSCRs Enhance Protein Production

Previously, we investigated the function of ALREX-promoting SSCRs in mediating mRNA localization of the *fushi tarazu* mRNA (*ftz*), a reporter transcript that has been used to study mRNA splicing [11] and nuclear export [6],[12],[13]. We also found that ALREX-promoting SSCRs promote the translation of the *ftz* reporter mRNA into protein (AFP, unpublished observations). However, these initial observations were uninformative as they could not determine whether ALREX-promoting SSCRs contain an RNA element that potentiates protein production in a manner independent of codon effects. Indeed, there is a correlation between (1) the frequency with which a codon is present in the genome (i.e., “codon usage”), (2) the amount of cognate tRNA that is present in the cytoplasm, and (3) the number of copies of that tRNA gene [14]–[16].

**Figure 1:**
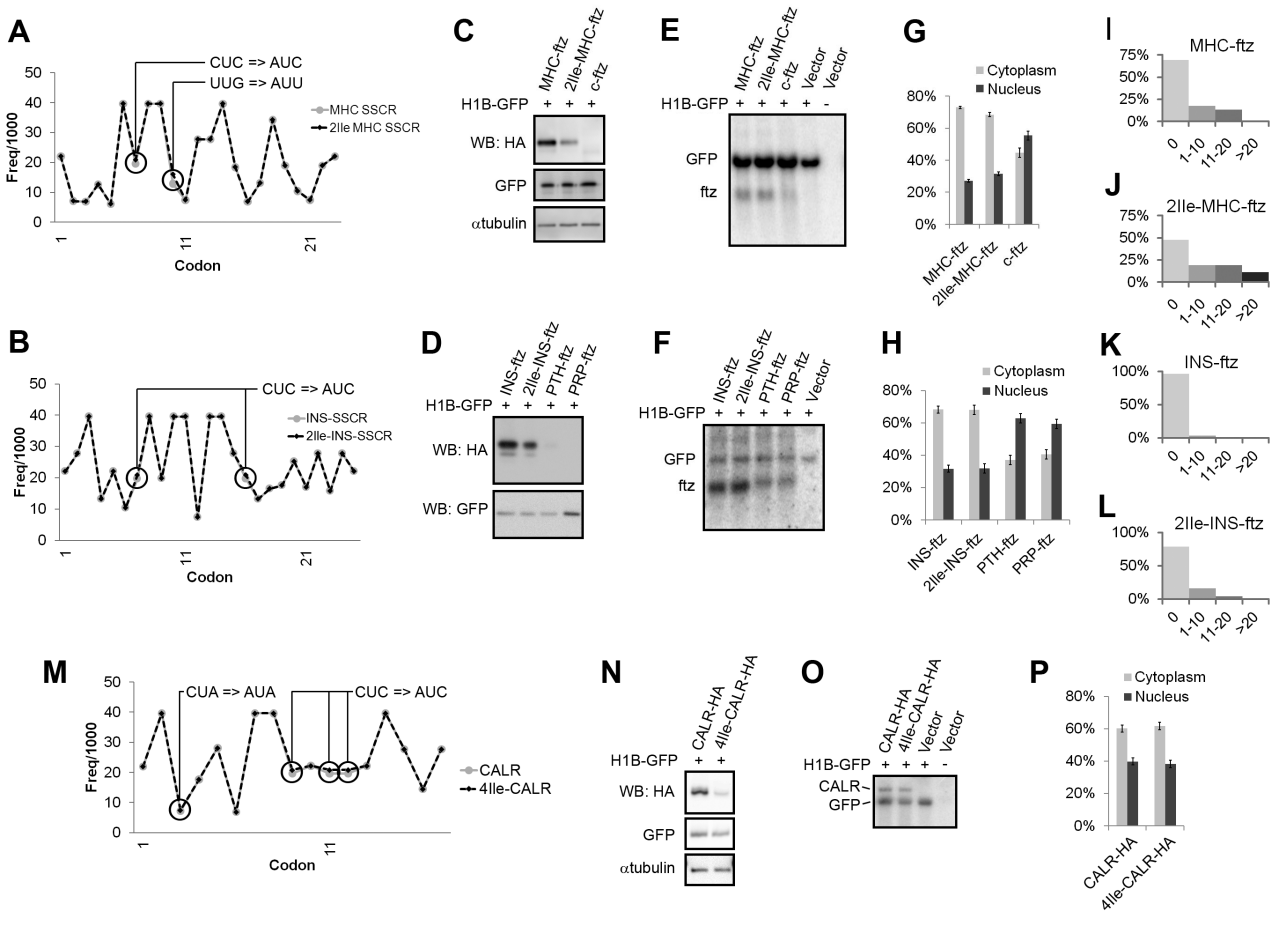
ALREX-promoting SSCRs promote translation. (A) For each codon in the *MHC*, *2Ile-MHC* SSCRs (*x*-axis), the frequency with which it appears in the human genome per 1,000 codons (“Freq/1000”; *y*-axis) was plotted. Note that the two leucine-to-isoleucine mutations are circled. (B) Codon analysis of *Ins*, *2Ile-Ins* SSCRs as in (A). (C–L) U2OS cells were co-transfected with plasmids that contained various versions of the *ftz* gene, and a second plasmid that either contained (“+”) or lacked (“−”) the *H1B-GFP* gene. As a control, cells were transfected with a plasmid lacking the *ftz* gene (“Vector”). The cells were then analyzed 18–24 h post-transfection. (C–D) Cell lysates were separated by SDS-PAGE and were probed with antibodies against the HA epitope, GFP, and α-tubulin. (E–F) RNA was extracted from the cell lysates, separated on a denaturing agarose gel, and analyzed by Northern blotting using [^32^P]-labeled probes directed against *ftz* and *GFP* transcripts. (G–H) The percentage of total mRNA found in the cytoplasm and nucleus as determined by the distribution of *ftz* mRNA by FISH staining. Each bar represents the average and standard error between three separate experiments (each experiment consisting of the average of at least 30 cells). (I–L) For cells expressing the indicated version of *ftz*, the percentage of cells (*y*-axis) with various numbers of SGs (*x*-axis) was plotted. Note that the SGs were detected by Tia1 immunostaining, but almost always contained an enrichment in *ftz* mRNA. For each graph, >160 cells (I–J) and >580 cells (K–L) were analyzed. Note that while >15% of *2Ile-INS-ftz* expressing cells had SGs, this number dropped to <1% in cells expressing *INS-ftz*. (M) For the first 17 codons (*x*-axis) in the wild type and 4Ile mutant *CALR* gene, the frequency with which it appears in the human genome per 1,000 codons (*y*-axis). Note that the four leucine-to-isoleucine mutations in *4Ile-CALR* are circled. (N–P) U2OS cells were co-transfected with plasmids that contained either version of the HA-tagged *CALR* gene, and a second plasmid that either contained (“+”) or lacked (“−”) the *H1B-GFP* gene. As a control, cells were transfected with a plasmid lacking the *CALR* gene (“Vector”). The cells were analyzed 18–24 h post-transfection. (N) Cell lysates were separated by SDS-PAGE and were probed with antibodies against the HA epitope, GFP, and α-tubulin. (O) RNA was extracted from the cell lysates, separated on a denaturing gel and analyzed by Northern blotting analysis using [^32^P]-labeled probes directed against *CALR* and *GFP* RNA. (P) The percentage of total mRNA found in the cytoplasm and nucleus as determined by the distribution of *CALR* mRNA by FISH staining.

To disrupt features that promote ALREX, such as adenine depletion, while simultaneously controlling for codon usage effects, we altered constructs that had ALREX-promoting SSCRs by mutating leucine codons so that they now encoded isoleucine. While leucine codons are relatively adenine-poor, all of the isoleucine codons contain at least one adenine. Moreover, isoleucine and leucine have almost the same hydrophobicity and the substitution of one for the other is unlikely to alter the strength of the encoded signal sequence [17]–[19]. We chose to alter two constructs, *MHC-ftz* and *insulin* (*INS*)-*ftz*, which contain ALREX-promoting SSCRs derived from the mouse major histocompatibility *H2kb* gene and the human *insulin* gene, so that each had two leucine-to-isoleucine mutations (creating the *2Ile-MHC-ftz* and *2Ile-INS-ftz* constructs; see Figure 1A, 1B, and Table S1). In each construct, the substituted codons had similar usage frequencies as the original ones (Figure 1A and 1B) [20]. We then transfected cells with plasmids containing these various *ftz* constructs and monitored ftz protein production 18–24 h later. Note that the *ftz* reporter contains sequences that code for HA and FLAG epitopes, as described previously [6], and can be used to detect the protein by immunoblot. To control for transfection efficiency and gel loading, we co-transfected the cells with plasmids containing the *Histone 1B (H1B)-GFP* gene, and immunoblotted for green fluorescent protein (GFP) and α-tubulin.

We found that cells expressing *MHC-ftz* mRNA produced about twice as much ftz protein when compared to those expressing *2Ile-MHC-ftz* mRNA (Figure 1C). We obtained the same result when we compared *INS-ftz* to *2Ile-INS-ftz* (Figure 1D). Strikingly, the total steady state levels (Figure 1E and 1F) and cytoplasmic/nuclear distributions (Figure 1G and 1H) of the two mutant mRNAs were almost identical to their wild-type counterparts. In contrast, no protein could be detected when the ftz construct lacked an SSCR (*c-ftz*) (Figure 1C), and very little protein was expressed from the *parathyroid hormone* (*PTH*)-*ftz* and *prion protein* (*PRP*)-*ftz* constructs (Figure 1D; Table 1), which contain SSCRs derived from the *parathyroid hormone* and *prion protein* genes. Both of these genes have an intron in their 5′ UTR, and as we previously documented, their SSCRs are adenine-rich (Table S1), and lack ALREX activity (Figure 1H; Table 1) [7]. Interestingly, these two mRNAs migrated more slowly, when compared to *INS-ftz* on a denaturing gel (Figure 1F). This last observation suggests that mRNAs with weak ALREX activity have longer poly(A)-tails and is consistent with reports that poorly exported mRNAs are hyper-polyadenylated [21],[22].

**Table 1:** Analysis of various SSCR-containing ftz constructs.

	Protein Levels		mRNA Levels		Fraction of the Total mRNA in the Cytoplasm	
	Avg	±	Avg	±	Avg	±
INS-ftz	1.00	0.00	1.00	0.00	0.68	0.01
2Ile-INS-ftz	0.63	0.07	1.01	0.03	0.68	0.02
PTH-ftz	0.10	0.06	1.01	0.40	0.38	0.02
PRP-ftz	0.01	0.00	0.90	0.45	0.41	0.01

Each data point consists of the average and standard error of three independent experiments. Protein levels were determined by densitometry analysis of immunoblots, mRNA levels were determined by densitometry analysis of northern blots, and the fraction of the total mRNA was determined as in Figure 1G and 1H. In each experiment, the protein and mRNA levels were normalized to that of *INS-ftz*.

The difference in protein levels between cells expressing reporter genes with normal and mutant ALREX-promoting SSCRs could potentially have been caused by alterations in the translocation, processing or steady state level of the resulting proteins. However, all of the detectable translational products, for both *MHC-ftz* and *2Ile-MHC-ftz*, were glycosylated indicating that both were efficiently translocated into the endoplasmic reticulum (ER) lumen (Figure S1A). This is not surprising as signal sequences containing either leucine or isoleucine serve equally well to promote translocation [17]. Moreover, since the various protein products differ only in their signal sequence, which is cleaved (Figure S1A–S1C), the various SSCR-containing *ftz* constructs all produce the exact same final processed polypeptide. In agreement with this, these two protein products had similar half-lives (Figure S1D–S1F). Thus the final difference in protein levels was not due to changes in translocation, processing, or protein stability.

In support of the idea that the mutations inhibit translation, SG formation was up-regulated in cells expressing the mutant forms of ftz as compared to those expressing their wild-type counterparts (Figure 1I–1L). When controlling for transfection efficiency, mRNA distribution and mRNA levels, 54% (±6%, *p*<0.00001) less protein was produced from *2Ile-MHC-ftz* than from *MHC-ftz* mRNA. Similar results were obtained for *INS-ftz* and *2Ile-INS-ftz* (Table 1).

In conclusion, these results strongly suggest that ALREX-promoting SSCRs act as an RNA element that enhances protein production from a reporter mRNA. In addition, our data indicate that the incorporation of a few silent adenine mutations has a much more dramatic impact on translation (Figure 1C and 1D) than on mRNA export (Figure 1G and 1H). This finding is consistent with our previous observations that many silent adenines had to be incorporated into ALREX-promoting SSCRs before we could observe effects on mRNA export [6],[7].

### ALREX-Promoting SSCRs Stimulate the Production of a Natural Protein

Next we monitored the production of calreticulin (CALR), a protein chaperone that resides in the lumen of the ER [23]. The *CALR* gene lacks a 5′ UTR intron and has an SSCR that is highly depleted of adenines. Within the first 55 nucleotides of the human *CALR* ORF, there is a tract of 43 nucleotides that lacks adenine, which is above average for the typical human SSCR [6].

To detect the expression of this protein, we inserted a sequence that codes for HA epitope into the 3′ end of the ORF. We also created a mutant form of the *CALR-HA* gene where four leucine codons were substituted for isoleucine codons that had similar usage frequencies (*4Ile-CALR-HA*; Figure 1M; Table S1). In agreement with our *ftz* reporter construct results, cells transfected with plasmids containing the *CALR-HA* cDNA gene produced more protein than those transfected with *4Ile-CALR-HA* (Figure 1N). Since the two protein products differ only in their signal sequences, which are cleaved during processing, we could again rule out that the difference in levels was due to changes in protein stability. Furthermore, we could also rule out that the change in protein production was due to differences in the level (Figure 1O) or cytoplasmic/nuclear distribution (Figure 1P) of *CALR-HA* mRNA.

From these experiments we conclude that ALREX-promoting SSCRs can potentiate the translation of a natural mRNA. When controlling for transfection efficiency, mRNA distribution and mRNA levels, 57% (±13%, *p*<0.005) less protein was produced from *4Ile-CALR-HA* than from *CALR-HA* mRNA. Confirming our results with the *ftz* reporter construct, we again observed that the disruption of the ALREX-element had a more pronounced effect on translation than on mRNA export.

### ALREX-Promoting Elements Require a Nuclear Factor in order to Stimulate ftz Protein Synthesis

**Figure 2:**
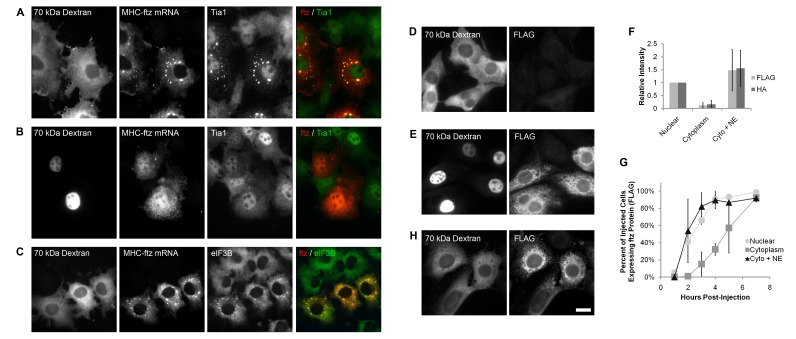
ALREX-promoting elements require a nuclear factor in order to stimulate ftz protein synthesis. (A–C) The cytoplasm (A,C) or nuclei (B) of COS-7 cells were microinjected with in vitro synthesized, capped, and polyadenylated *MHC-ftz* mRNA and FITC-labeled 70 kDa dextran. After 2 h cells were fixed and stained by FISH using probes against *ftz* mRNA and by immunofluorescence using antibodies directed against Tia1 (A–B) or eIF3B (C). Each row represents a single field of cells. Note that in cytoplasmically injected cells the *MHC-ftz* mRNA accumulated in cytoplasmic aggregates that are enriched in Tia1 and eIF3B (see overlays in (A and C)). (D–E, H) NIH-3T3 cells were microinjected with FITC-labeled 70 kDa dextran and in vitro synthesized, capped, and polyadenylated *MHC-ftz* mRNA, which was either untreated (D–E) or pre-incubated with HeLa nuclear extract (H). The cells were incubated for 3 h then fixed and stained by immunofluorescence using antibodies directed against the FLAG epitope. Each row represents a single field of cells that were microinjected into the cytoplasm (D,H) or nucleus (E) as determined by the distribution of 70 kDa dextran. Note that when *MHC-ftz* mRNA is introduced directly into the cytoplasm it is translated only if it was pre-incubated with nuclear extract. Scale bar = 20 µM. (F–G) NIH-3T3 cells were microinjected with *MHC-ftz* mRNA and FITC-labeled 70 kD dextran, alone or with HeLa nuclear extract (“+NE”), in the nucleus or cytoplasm. Cells were fixed, then immunostained for either FLAG (F–G) or HA (F) epitopes. (F) The total integrated intensity of FLAG and HA immunofluorescence was quantified for cells, 3 h post-injection. (G) The percentage of microinjected cells expressing ftz protein, as measured by the appearance of FLAG immunofluorescence, was quantified for each time point post-injection. Each data point/bar in (F–G) consists of an average of three experiments, each of which consisting of >100 cells. Error bars represent the standard deviation between experiments.

When the translation of *MHC-ftz* and *2-Ile-MHC-ftz* was monitored using an in vitro reticulocyte extract, both mRNAs produced the same amount of protein (Figure S1C). From this result we concluded that there is no inherent difference in the translatability of these two mRNAs and that reticulocyte lysate, which is derived from enucleated cells, may lack factors that are required for the efficient translation of transcripts harboring ALREX-elements. Indeed, ALREX-elements are likely to associate with nuclear factors that promote mRNA export and then accompany the transcript to the cytoplasm where they may help to potentiate translation. To test this idea, we microinjected in vitro synthesized and polyadenylated *MHC-ftz* mRNA into either the cytoplasm or nucleus of cells, and after various time points, we visualized *ftz* mRNA by fluorescence in situ hybridization (FISH). Cytoplasmic or nuclear microinjection was confirmed by co-injecting fluorescently labeled 70 kDa dextran, which is too large to passively cross the nuclear pores. When injected into the cytoplasm, but not the nucleus, *MHC-ftz* mRNA accumulated in aggregates that were positive for Tia1, a general marker for SGs (Figure 2A–2B) [24]. In general, SGs are thought to form under a variety of conditions, including the accumulation of mRNA with defective or incompletely assembled translation initiation complexes [25],[26]. Since these SGs were also enriched in eIF3B (Figure 2C), a translation initiation factor, it is likely that the mRNAs that were injected into the cytoplasm assembled incomplete translation-initiation complexes, rather than simply aggregating non-specifically. Interestingly, mRNA that was injected into the cytoplasm appeared to be stable, as the FISH signal remained strong for up to 9 h post-injection (unpublished data).

We next investigated whether the injected *MHC-ftz* mRNAs were translated. Since too few cells are microinjected in a single experiment to allow for biochemical analysis, we instead monitored the expression of protein by immunofluorescence against the HA and FLAG epitopes, which are encoded within the *ftz* ORF. When *MHC-ftz* mRNA was microinjected into the cytoplasm, few of those cells produced detectable levels of ftz protein (Figure 2D). In contrast, most cells whose nuclei were injected with the same mRNA expressed protein after about 3 h (Figure 2E). As expected, all expressed protein co-localized with the ER marker Trapα (unpublished data), as previously reported [6]. When we quantified the total intensity of the FLAG and HA immunostain signals 3 h post-injection, there was a 10- to 15-fold increase in protein production when the mRNA was microinjected in the nucleus (Figure 2F). Interestingly, when we monitored this expression over time, cells whose cytoplasm was injected with *MHC-ftz* mRNAs eventually expressed protein, but only after a 2–3 h delay when compared to those that received *MHC-ftz* mRNAs in the nucleus (Figure 2G). We speculated that the delay in expression was due to the fact that over time these mRNAs were slowly accumulating a factor that cycled between the nucleus and the cytoplasm. Remarkably, when *MHC-ftz* mRNA was pre-incubated with HeLa nuclear extract and then injected into the cytoplasm, efficient protein production was restored (Figure 2F–2H). When *c-ftz* mRNA was injected either into the nucleus or cytoplasm, with or without nuclear extract, no translational product was ever detected (unpublished data). These observations suggest that ALREX-promoting elements potentiate translation by recruiting one or more nuclear factors to the translational start site.

### Identification of Nuclear Proteins That Associate with ALREX-Promoting Elements

**Figure 3:**
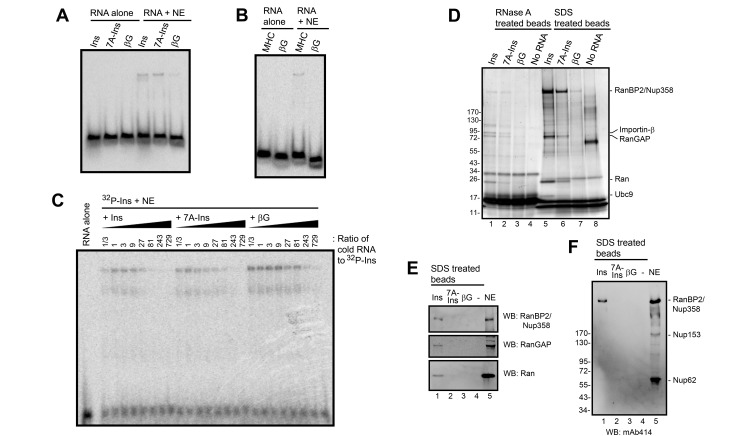
Identification of ALREX-element associating proteins from HeLa nuclear extract. (A–B) Various [^32^P]-labeled RNA fragments, either 82 (*Ins* SSCR, *7A-Ins* SSCR, and the 5′ end of the βG ORF) or 86 (*MHC* SSCR) nucleotides long, were incubated with or without HeLa nuclear extract (“NE”) and separated on a non-denaturing polyacrylamide gel and visualized by autoradiography. (C) [^32^P]-labeled *Ins* SSCR RNA was mixed with increasing amounts of various unlabeled RNA fragments, then incubated with NE. The reactions were separated on a non-denaturing polyacrylamide gel and visualized by autoradiography. (D–F) Streptavidin-coated magnetic beads, bound with either various biotinylated RNAs (each 76 nucleotides long) or without any RNA (“No RNA”), were used to isolate proteins from HeLa nuclear extract. Proteins were eluted by first treating the beads with RNase A (D, lanes 1–4), followed by denaturation in SDS at 90°C for 5 min (D, lanes 5–8). (D) Silver stained gel of the eluted proteins. Bands identified by mass spectrometry are indicated on the right. (E–F) Eluted proteins and NE were analyzed by immunoblots using an antibody that recognizes, RanBP2, RanGAP1, Ran, or FG-repeat Nucleoporin proteins (mAb414).

Since HeLa nuclear extract appeared to potentiate translation, and would also be enriched in putative nuclear export factors, we wanted to determine whether any proteins in these extracts directly associate with ALREX-promoting elements. Using electrophoretic mobility shift assays (EMSAs), we observed the formation of a complex between nuclear extract factors and [^32^P]-labeled RNA fragments from the *Ins* and *MHC* SSCRs (Figure 3A and 3B). Complex formation was detected when we tested a mutant form of the *Ins* SSCR containing seven silent adenine substitutions (*7A-Ins*; Figure 3A; see Table S1 for the sequence of this mutant). In contrast, very little complex was formed with an RNA fragment derived from the beginning of the *β-globin* (*βG*) ORF (Figure 3A and 3B), which does not promote export [27]. To test for specificity, we formed complexes between HeLa nuclear extracts and the *Ins* SSCR as in Figure 3A, but in the presence of cold competitor RNA. Unlabeled *Ins* SSCR was a more effective competitor than either the *7A-Ins* mutant or the *βG* RNA fragment (Figure 3C). From these experiments we concluded that one or more factors in HeLa nuclear extracts specifically associate with ALREX-promoting elements. Our data indicate that the addition of adenines decreases, but does not abolish, the ability of HeLa factors to interact with ALREX-promoting elements. This result is consistent with the observation that adenine substitutions only partially inhibit ALREX (Figure 1 G and 1H) [6],[7].

To isolate these interacting proteins from HeLa nuclear extracts we incubated these with biotinylated RNA fragments that were immobilized on streptavidin-conjugated magnetic beads. Bound proteins were eluted by treating the beads with RNAse A (Figure 3D, lanes 1–4). Since RNA-binding factors can protect the biotinylated transcripts from digestion, we then treated the beads with SDS at high temperature to denature any remaining proteins (Figure 3D, lanes 5–8). Several *Ins* SSCR-interacting factors were detected by silver stain. Importantly, these proteins were less abundant when the purification was performed with the *7A-Ins* mutant RNA, and almost completely absent with *βG* RNA. Note that these proteins also associated specifically with the *Ins* SSCR under less stringent conditions, but were partially obscured by non-specific RNA-binding proteins (Figure S2). Since the nonspecific proteins bound equally well to beads with either the *Ins* SSCR, the *7A-Ins* mutant, and *βG* RNAs, but not to the beads alone, we are somewhat confident that equal amounts of RNA were used in all pulldowns. Bands were excised and identified using mass spectrometry as being RanBP2/Nup358 (henceforth termed RanBP2), Importinβ, RanGAP1, Ran, and Ubc9. When proteins from RNAse A and SDS eluates were precipitated with trichloroacetate and analyzed by mass spectroscopy, we could also detect TAP, RCC1, and Importinα in the *Ins* purified fraction, but not the other fractions. Since these proteins could not be readily visualized on a silver-stained gel (Figure 3D), we believe that their binding to the *Ins* SSCR is either very weak or indirect. We also detected a large number of SUMO peptides in the trichloroacetate-precipitated fraction. Interestingly, RanBP2 is a known E3 SUMO ligase, which transfers SUMO from Ubc9 (an E2 SUMO ligase) to substrates such as RanGAP1 [28]. Moreover RanBP2, which is present on the cytoplasmic face of the nuclear pore, forms a stable complex with Ubc9 and sumoylated RanGAP1 [29],[30]. RanBP2 also interacts with most of the other identified proteins, including Ran [31],[32], TAP [33],[34], and Importinβ [35].

To validate our findings, we assessed the association of HeLa extract proteins to the RNA-coated beads by immunoblot. To ensure that the binding was specific, we washed the beads extensively with high salt buffer. Indeed, RanBP2, RanGAP1, and Ran bound exclusively to *Ins*, but not *7A-Ins*, *βG* RNAs, or to the beads alone (Figure 3E). When the fractions were probed with mAb414, an antibody that recognizes several nucleoporins [36],[37], only RanBP2 was detected in the *Ins* SSCR pulldown (Figure 3F). In contrast, Nup62 and Nup153 failed to associate with the RNA (Figure 3F, lane 1) despite the fact that they are present in this extract (Figure 3F, lane 5).

From these results we conclude that the *Ins* SSCR interacts with RanBP2 and several RanBP2-associated proteins.

### The Zinc Finger Repeats of RanBP2 Interact Directly with the Ins SSCR

**Figure 4:**
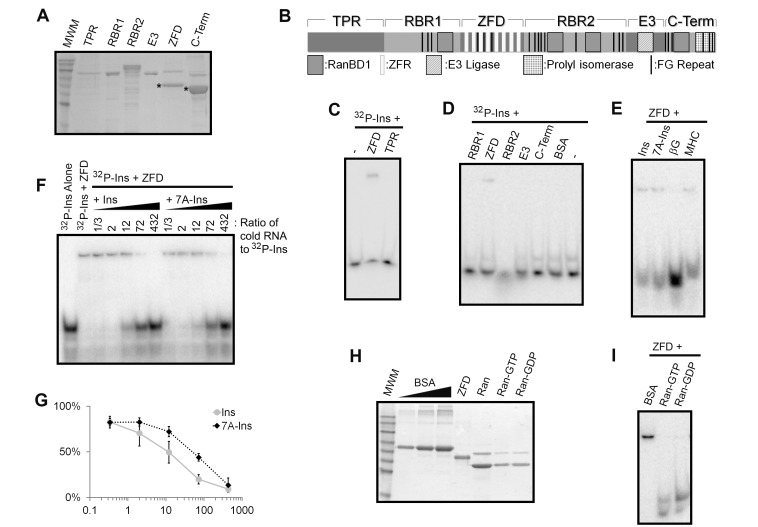
The RanBP2 zinc fingers directly interact with ALREX-elements. (A) Various His-tagged (TPR, tricopeptide repeat region; RBR1/2, ran binding domain-containing regions 1/2; C-Term, carboxy-terminal domain) or GST-tagged (E3, E3 SUMO ligase domain) fragments of the RanBP2 gene were expressed in bacteria and purified. The proteins were separated by SDS-PAGE and stained with Coomassie blue. In cases where multiple bands appear, proteins that are detected on anti-His-tag immunoblots are denoted by asterisks. (B) Domain structure of RanBP2. Note that this protein contains four type 1 Ran binding domains (RanBD1), eight ZFRs, an E3 ligase domain, a cis-trans prolyl-isomerase domain, and several FG repeats. (C–D) [^32^P]-labeled *Ins* SSCR RNA was incubated with purified proteins, separated on a non-denaturing polyacrylamide gel, and then visualized by autoradiography. Note that the ZFD forms a complex with the *Ins* RNA. (E) Various [^32^P]-labeled RNA fragments were incubated with the ZFD protein, separated on a non-denaturing polyacrylamide gel, and then visualized by autoradiography. (F) [^32^P]-labeled *Ins* SSCR RNA was incubated with or without the ZFD protein fragment in the absence or presence of various unlabeled RNAs. The complexes were separated on a non-denaturing polyacrylamide gel and then visualized by autoradiography. (G) Graph of the amount of [^32^P] *INS*-containing complexes (*y*-axis) in the presence of various amounts of unlabeled RNAs (*x*-axis). Each point is the average and standard deviation of two independent experiments. (H) Coomassie stain of bacterially expressed ZFD and GST-tagged Ran before and after loading with GTP or GDP. In order to estimate the levels of Ran and ZFD proteins, various amounts of BSA were also loaded on the gel. (I) ZFD was preincubated with either BSA or Ran loaded with GDP or GTP, then incubated with [^32^P]-labeled *Ins* SSCR RNA. The complexes were separated on a non-denaturing polyacrylamide gel and then visualized by autoradiography.

Since all of the proteins that associated with the *Ins* SSCR are known to bind to RanBP2, it is highly probable that the RNA is making contact to one subunit of a large complex. We expressed and purified five His-tagged, and one GST-tagged, fragments of RanBP2. Each fragment corresponded to a different region of the protein (Figure 4A and 4B, tagged proteins that react positively on an immunoblot with anti-His antibodies are denoted by asterisks). We found that the zinc finger domain (ZFD) fragment, which contains all eight of the RanBP2 ZFRs (see Figure 4B), was able to form a complex with [^32^P]-labeled *Ins* RNA by EMSA (Figure 4C and 4D). In contrast, none of the other fragments were able to form a complex (Figure 4C and 4D). In addition we did not detect binding between [^32^P]-labeled *Ins* SSCR and either purified bacterially expressed RanGAP1 or Importinβ alone, or in the presence of GTP-bound Ran (unpublished data). The ZFD also formed a complex with the *MHC* SSCR, but not the *βG* RNA (Figure 4E). Although, the mutant *7A-Ins* SSCR associated with purified ZFD (Figure 4E), this RNA showed reduced affinity as compared to wild-type *Ins* SSCR in competition assays (Figure 4F and 4G). From these results we conclude that RanBP2 has the ability to bind directly to the *Ins* and *MHC* SSCRs through its ZFRs.

### Ran and the *Ins* SSCR Interact with the ZFD in a Mutually Exclusive Manner

Interestingly, the eight ZFRs from RanBP2 are highly related to repeats found in Nup153 and ZRanB2. Indeed, the ZRanB2 zinc fingers bind directly to single-stranded RNA in a sequence-specific manner [38]. Although the zinc fingers from RanBP2 and Nup153 had not been previously reported to interact with RNA, they were shown to directly associate with both GDP- and GTP-bound Ran [39]. Interestingly, structural and biochemical analysis indicates that the RanBP2 and Nup153 zinc fingers interact with Ran using the same analogous surface that the ZRanB2 repeats employs to bind RNA [38],[39]. This raised the possibility that the *Ins* SSCR and Ran may compete for binding to the RanBP2 ZFRs. In agreement with this idea, GST-tagged Ran, loaded with either GTP or GDP (Figure 4H), effectively prevented the ZFD protein fragment from associating with the *Ins* SSCR (Figure 4I, lanes 2 and 3). In contrast, BSA, which does not associate with the *Ins* SSCR (see Figure 4D), had no effect on complex formation (Figure 4I, lane 1). From these results we conclude that the binding of the *Ins* SSCR and Ran to ZFD is mutually exclusive. Our results are consistent with the idea that the ZFD uses a similar surface to bind to both molecules, although we cannot exclude the possibility that Ran-binding may induce a conformational change in the ZFD that ultimately alters a different RNA binding-site.

### The Number of RanBP2 Zinc Fingers Correlates with the Average Length of SSCR-Specific Adenine-Less Tracts across Metazoans

**Figure 5:**
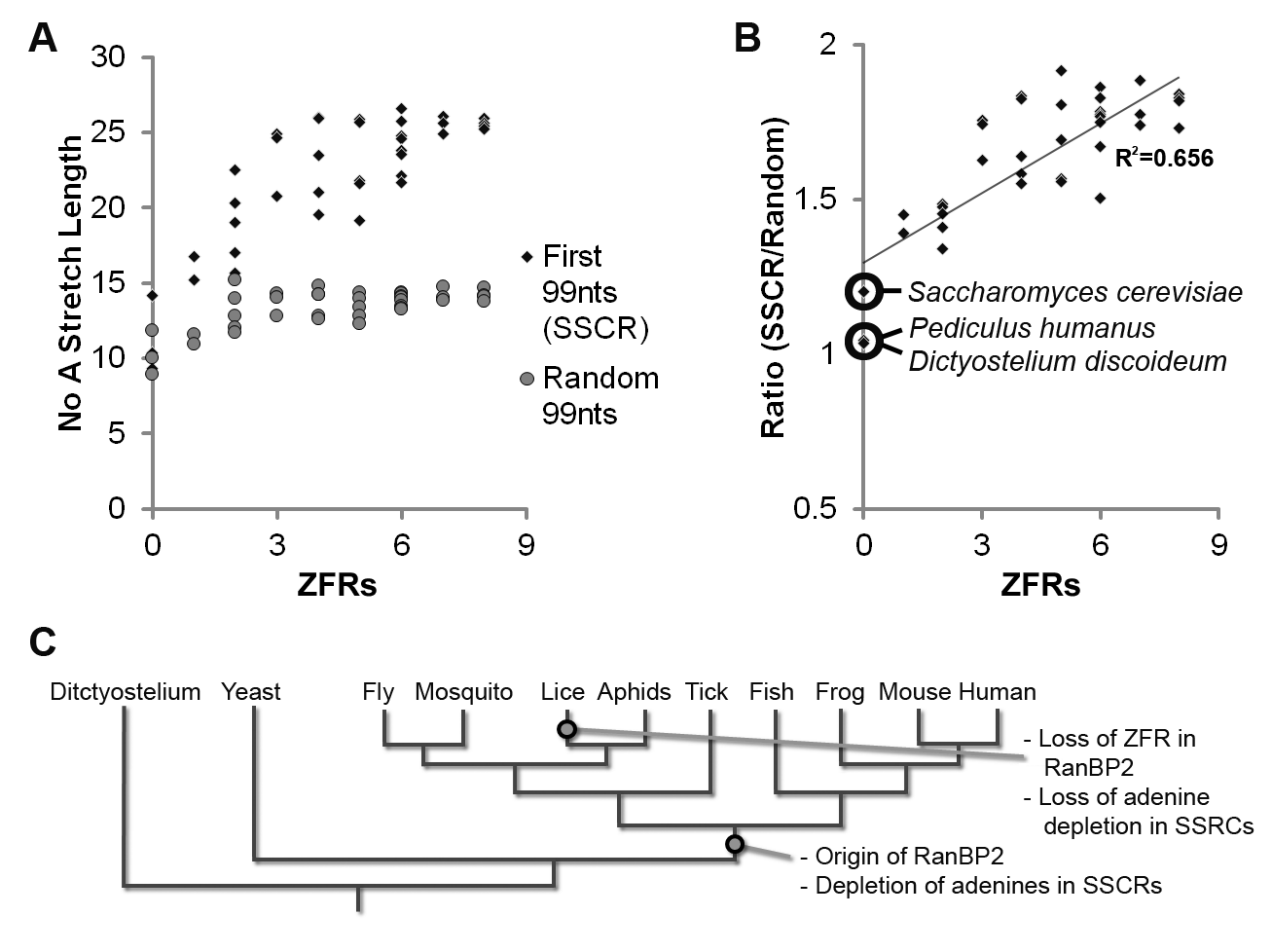
Adenine depletion in SSCRs correlates with the number of RanBP2 zinc fingers across metazoans. (A–B) For each organism, the ORFs from at least 500 genes encoding secreted proteins were analyzed (for individual values see Table S2). The average length of the longest adenine-less track within the first 99 nucleotides or a random stretch of 99 nucleotides was analyzed. (A) For each organism, the average adenine-less track lengths (*y*-axis) were plotted against the number of ZFRs (*x*-axis) in the RanBP2 gene. (B) The ratio of adenine-less track lengths in the first 99 nucleotides versus a random stretch of 99 nucleotides (*y*-axis) was plotted against the number of ZFRs in the RanBP2 (*x*-axis) within each species. A trend line and the corresponding coefficient of determination (R^2^) was also included. Note that for one metazoan species, *P. humanus* (human head lice), there is neither adenine depletion nor ZFRs within RanBP2. Also note that non-metazoans (*D. discoideum* and *S. cerevisiae*) lack both the SSCR-specific adenine depletion and the RanBP2 gene. (C) A phylogenetic tree depicting the likely loss of both adenine depletion and RanBP2 zinc fingers in the lineage that gave rise to *P. humanus*.

Adenine depletion is a hallmark of ALREX-promoting elements [6],[7]. We previously capitalized on this fact to estimate the size of ALREX-promoting elements in various organisms by measuring the longest tract of adenine-less sequence within the first 69 nucleotides of ORFs that encode signal sequences [6]. Indeed, the average length of adenine-less sequence per SSCR varied greatly between species [6]. We speculated that this variation between organisms might correlate with the number of zinc fingers present in their respective copy of RanBP2. To test this idea, we expanded our analysis to 36 different metazoans where we calculated the average length of the longest tract of adenine-less sequence within the first 99 nucleotides of the ORFs that encode signal sequences, and compared this to the number of zinc fingers encoded by that organism's *RanBP2* gene. As a control we computed the average length of the longest tract of adenine-less sequence in a stretch of 99 nucleotides selected randomly within each ORF. In addition we also analyzed genes that code for signal sequences from *Dictyostelium discoideum* and *Saccharomyces cerevisiae*, two organisms that do not contain any known RanBP2 orthologs. Overall, we observed a general correlation between the number of RanBP2 ZFRs and the length of adenine-less tracts in SSCRs (Figure 5A and 5B; Table S2). Critically, SSCRs from *D. discoideum* and *S. cerevisiae* did not contain long adenine-less tracts when compared to control sequences (Figure 5A and 5B). Since RanBP2 has yet to have been documented outside of metazoans, our data suggest that the selective pressure necessary to effectively deplete adenines from SSCRs arose only after the appearance of RanBP2 at the base of the metazoan tree (Figure 5C). Even more interesting is the case of *Pediculus humanus* (commonly referred to as the human head louse), which did not display any significant increase in length of adenine-less tracts in their SSCRs and whose RanBP2 gene lacked ZFRs. In contrast, six other invertebrates (including aphids) had both a significant increase in the length of the adenine-less tracts in their SSCRs and versions of RanBP2 that contained ZFRs. Thus, it is likely that the lineage from which *P. humanus* arose, lost both features relatively recently (i.e., after this lineage had diverged from aphids, Figure 5C).

This analysis indicated that these two features (i.e., zinc fingers within RanBP2 and SSCR-specific adenine depletion) not only correlated, but are either both present or absent in all organisms analyzed thus far. We thus concluded that adenine-less tracts and the zinc fingers of RanBP2 display an evolutionary relationship towards each other. This finding strongly indicates that a major role of the RanBP2 ZFRs is to recognize adenine-less tracts in ALREX-promoting SSCRs.

### RanBP2 Is Required for the Potentiation of Translation by ALREX-Promoting SSCRs

**Figure 6:**
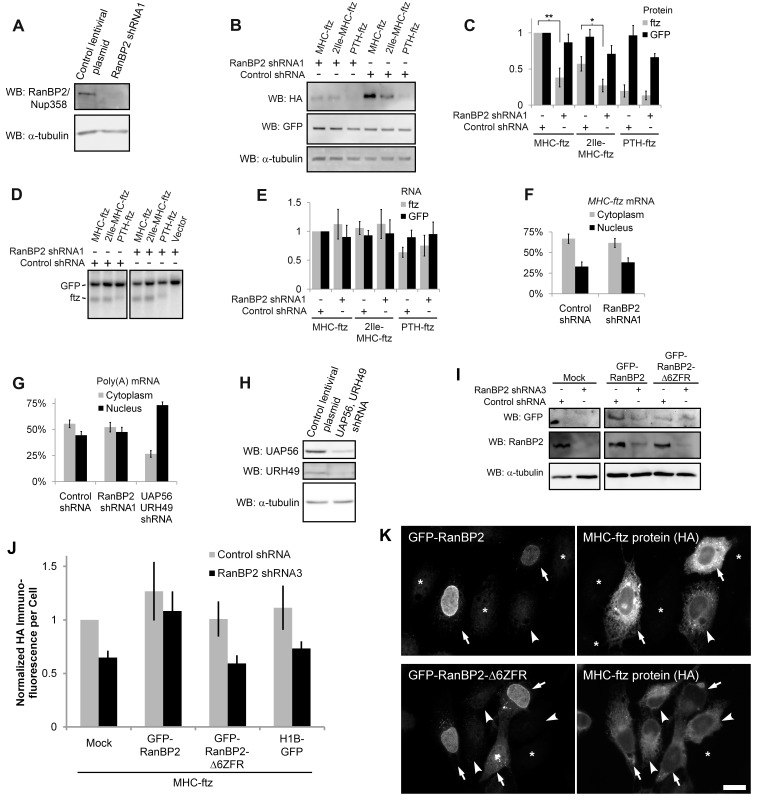
RanBP2 is required for ALREX-mediated translation enhancement. (A) U2OS cells were infected with lentivirus containing shRNA1 directed against RanBP2 or control virus. Four days post-infection, cell lysates were collected, separated by SDS-PAGE on a 6% acrylamide gel, and immunostained for RanBP2 or α-tubulin. (B–E) U2OS cells were infected with lentivirus that delivered shRNA1 against RanBP2 or control virus. Three days post-infection, cells were transfected with plasmids containing various *ftz* constructs and *H1B-GFP*. 18–24 h post-transfection the level of protein was analyzed by immunoblot (B, quantification in C) and mRNA by northern blot (D, quantification in E). Each bar represents the average and standard error of six independent experiments. *, *p*<0.025; **, *p*<0.01. (F) Three days post-infection, U2OS cells were transfected with plasmid containing *MHC-ftz*. 18–24 hr post-transfection the amounts of *MHC-ftz* mRNA found in the cytoplasm and nucleus was determined by FISH. One bar represents an average of three experiments, each of which consists of 20–30 cells. Error bars represent the standard deviation between the three experiments. (G) U2OS cells were treated with various lentiviruses. Four days post-infection, cells were fixed and stained for poly(A) mRNA by FISH. The average levels of poly(A) mRNA in the cytoplasm or nucleus were plotted. One bar represents an average of three experiments, each of which consists of 20–30 cells. Error bars represent the standard deviation between the three experiments. (H) UAP56 and URH49 were simultaneously depleted in U2OS cells by lentiviral mediated delivery of shRNAs. Four days post-infection, cell lysates collected, separated by SDS-PAGE, and immunostained for these two proteins and for α-tubulin. (I–K) U2OS cells were infected with lentivirus containing shRNA directed against the 3′ UTR of RanBP2 (shRNA3) or control virus. (I) Three days post-infection, cells were transfected with plasmids containing GFP-RanBP2, or GFP-RanBP2Δ6ZFR or without plasmid (“Mock”). After allowing expression for 48 h, cell lysates were collected and detected by immunoblot using antibodies against GFP, RanBP2, and α-tubulin. Note that the GFP-RanBP2 constructs do not contain the endogenous UTRs and are resistant to depletion by shRNA3. (J–K) Three days post-infection, cells were co-transfected with plasmids containing MHC-ftz and either GFP-RanBP2, GFP-RanBP2Δ6ZFR, or H1B-GFP and allowed to express for 48 h. Cells were immunostained using anti-GFP and anti-HA primary antibodies, and the appropriate fluorescent secondary antibodies. (J) For cells co-expressing GFP and MHC-ftz, the HA-immunofluorescence intensity was tabulated, averaged, and then normalized to the level of expression in mock, control shRNA-treated cells expressing MHC-ftz. Each bar represents the average and standard error between four independent experiments, each of which consists of 30–50 cells. Examples of cells depleted of endogenous RanBP2 with shRNA3 and expressing various GFP-RanBP2 constructs and MHC-ftz are shown in (K). Each row is a single field co-immunostained for GFP and HA. Note that cells expressing GFP-RanBP2 (top row, arrows), express higher levels of MHC-ftz than neighboring cells that express MHC-ftz alone (top row, arrowheads). In contrast, cells expressing GFP-RanBP2Δ6ZFR (bottom row, arrows) express about as much MHC-ftz protein as cells expressing MHC-ftz alone (bottom row, arrowheads). Un-transfected cells are denoted by asterisks. Scale bar = 20 µM.

We next examined whether RanBP2 was required for the efficient translation of mRNAs containing ALREX-promoting SSCRs. To accomplish this we transiently transfected reporter constructs into U2OS cells treated with lentiviral-delivered shRNAs directed against RanBP2. We found that cells depleted of RanBP2 (Figure 6A) did not efficiently produce MHC-ftz protein when compared to control cells (Figures 6B and 6C). In contrast, the expression of PTH-ftz and H1B-GFP protein was relatively unaffected (Figures 6B and 6C). Interestingly, we also observed a drop in the production of 2Ile-MHC-ftz. This result is not surprising when considering that the RanBP2 zinc-finger repeats bind to ALREX-promoting sequences that had adenine mutations, albeit at a lower level than wild-type sequences (see Figure 4F and 4G). The decrease in MHC-ftz and 2Ile-MHC-ftz protein synthesis could not be attributable to changes in the total level *ftz* mRNAs, which remained unaffected (Figures 6D and 6E), or to changes in the distribution of *MHC-ftz* mRNA between the cytoplasm and the nucleus (Figure 6F).

This last finding was quite surprising given the fact that RanBP2 was shown to be required for the efficient nuclear export of bulk mRNA in insect and mouse cells [33],[40]. In contrast, it had also been reported that RanBP2 depletion had no effect on the distribution of poly(A) mRNA in the human HeLa cell line [41]. In line with this last experiment we found that the cytoplasmic/nuclear ratio of poly(A) mRNA was unaffected by the depletion of RanBP2 in human U2OS cells (Figure 6G). On the other hand, depletion of the two RNA helicases, UAP56 and URH49, by shRNA treatment (Figure 6H) caused a pronounced block in mRNA nuclear export (Figure 6G) as described previously [42]. Thus it is likely that RanBP2 plays at most a minor role in mRNA nuclear export in human cells. This might be due to the presence of RanBP2 paralogs, which appear to be primate specific [43]. Curiously, these paralogs lack the ZFRs and are thus unlikely to participate in ALREX-mediated events.

We next evaluated the production of protein from a more “natural” transcript. As expected, the depletion of RanBP2 also inhibited the production of CALR-HA, but not 4Ile-CALR-HA protein without a significant change in mRNA level (Figure S3A–S3C). To confirm these results, we depleted RanBP2 with a second shRNA construct (“RanBP2 shRNA2”; Figure S3D). In these cells we again observed a consistent reduction in the production of MHC-ftz and CALR-HA protein without a significant change in mRNA levels (Figure S3E–S3H). In contrast, the levels of PTH-ftz and H1B-GFP proteins were relatively unaffected. Finally we found that the depletion of RanBP2 using either of the two shRNAs inhibited the synthesis of INS-ftz protein (Figure S3I and S3J).

To ensure that our results were not due to some pleiotropic effect, we depleted RanBP2 with a third lentiviral-delivered shRNA (i.e., shRNA3) that is complementary to a region in the 3′ UTR. Three days after infection we rescued the knockdown cells by transfecting a plasmid that contains a GFP-RanBP2 construct that lacks the endogenous UTRs and thus was resistant to depletion (Figure 6I). In parallel we transfected control plasmids containing either a version of GFP-RanBP2 that lacks the last six zinc fingers (RanBP2Δ6ZFR) or H1B-GFP. With these various GFP vectors we co-transfected a plasmid containing *MHC-ftz*. Since, only a subset of the MHC-ftz expressing cells also co-expressed the GFP-RanBP2 constructs (Figure S4), we monitored individual cells for ftz protein levels by immunofluorescence against the HA epitope. Since GFP-RanBP2 expression was very low we also immunostained cells with an antibody against GFP. In cells transfected with *MHC-ftz* alone, RanBP2 depletion by shRNA3 resulted in a decrease in protein expression by approximately 2-fold (Figure 6J, “mock”). Co-expression of GFP-RanBP2 rescued the level of ftz protein, whereas co-expression of GFP-RanBP2Δ6ZFR, had no effect (Figure 6J). Note that even in the shRNA3-treated cells, those that express GFP-RanBP2, express much more ftz protein than neighboring cells that express ftz alone (Figure 6K, compare the level of ftz protein in the cells expressing GFP-RanBP2, which are denoted by arrows, to the cell expressing ftz alone, which is indicated by an arrowhead). In contrast, expression of GFP-RanBP2Δ6ZFR had no effect on ftz protein levels (Figure 6K).

**Figure 7:**
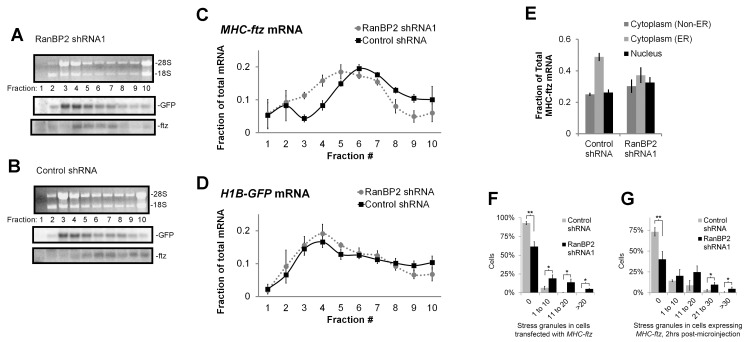
Depletion of RanBP2 shifts ***MHC-ftz*** **from polysomes to monosomes.** U2OS cells were infected with lentivirus that delivered shRNA1 against RanBP2 or control virus. (A–D) Three days post-infection, cells were transfected with plasmids containing *MHC-ftz* and *H1B-GFP*. 18–24 h post-transfection 0.6 ml of cell lysates were layered onto a 10.5 ml sucrose gradient (25%–45%) and centrifuged at 200,000 *g* for 2.5 h. Ten equal fractions were manually collected (the top of the gradient is on the left), RNA was purified and separated by electrophoresis on a denaturing agarose gel. The distributions of 18S and 28S rRNA were analyzed by ethidium bromide staining (see Figure S5 for quantification) and the distribution of *ftz* and *GFP* mRNA were analyzed by Northern blot (A–B). Note that monosomes are found predominantly in fractions 3–4. (C–D) The levels (*y*-axis) of the two mRNAs in each fraction (*x*-axis) were analyzed by densitometry analysis and plotted. Each data point is the average and standard error of three independent experiments. (E–F) Three days post-infection, cells were transfected with plasmids containing *MHC-ftz*, which was allowed to express for 18–24 h. (E) Cells were either immediately fixed to visualize cytoplasmic and nuclear mRNA, or briefly permeabilized with digitonin to remove all cytoplasmic mRNA that was not anchored to the ER, and then fixed as described previously [44],[67]. *MHC-ftz* mRNA was visualized by FISH staining and the fraction of fluorescence intensity associated with each compartment was calculated for at least 30 cells in a single experiment. Each bar represents the average and standard error between three separate experiments. (F) Transfected cells were fixed to visualize *MHC-ftz* mRNA by FISH and Tia-1 by immunofluorescence. The percentage of cells (*y*-axis) with various numbers of SGs (*x*-axis) was plotted. Each bar represents the average and standard error of three independent experiments. (G) Four days post-infection cells were microinjected with plasmids containing *MHC-ftz*. After 20 min further mRNA synthesis was halted by the addition of α-amanitin, and cells were then fixed 2 h later. Cells were stained for *MHC-ftz* mRNA and Tia-1 and SGs were tabulated as in (F). *, *p*<0.025; **, *p*<0.01.

We next examined the association of *MHC-ftz* with polysomes using sucrose gradient centrifugation. Consistent with our analysis of translational product, *MHC-ftz* mRNA was associated with fewer ribosomes in RanBP2-depleted cells (Figure 7A–7C). In contrast, the distributions of *H1B-GFP* mRNA (Figure 7A, 7B, and 7D) and ribosomal rRNA (Figures 7A,7B, and S5) in the gradients were relatively unaffected. Since active translation is required for the efficient localization of *MHC-ftz* mRNA to the ER [44], we next examined the distribution of this mRNA on this organelle. In order to accomplish this we extracted cells with digitonin, a detergent that selectively permeabilizes the plasma membrane and thus removes cytoplasmic mRNAs that are not anchored to the ER [44],[45]. By analyzing the level of *MHC-ftz* mRNA in unextracted and extracted cells by FISH, we calculated that the amount of ER-bound *MHC-ftz* mRNA was reduced in cells depleted of RanBP2 (Figure 7E). Finally we assessed the association of *MHC-ftz* mRNA with SGs by co-staining this transcript with Tia1. RanBP2-depleted cells transfected with plasmids encoding *MHC-ftz* mRNA had an increase in SG formation when compared to transfected control cells (Figure 7F). As seen previously, the vast majority of these SGs were enriched in *MHC-ftz* mRNA. To determine how fast these SGs formed, we microinjected plasmids into these cells and monitored the distribution of newly made mRNA levels. 20 min post injection we halted the further production of mRNA by adding α-amanatin and we then monitored the distribution of newly synthesized mRNA by FISH. Indeed, SGs appeared in RanBP2-depleted cells that expressed *MHC-ftz* mRNA after only 2 h post-injection (Figure 7G). Moreover the newly expressed mRNAs accumulated in these SGs. These observations are consistent with the idea that in RanBP2-depleted cells, there is a buildup of *MHC-ftz* mRNAs with defective translation-initiation complexes that very quickly aggregate into SGs [9].

From these results we conclude that RanBP2 is required for the potentiation of translation by ALREX-promoting SSCRs. Furthermore, in cells depleted of RanBP2 we observed a decrease in the amount of *MHC-ftz* transcripts that are associated with the ER and polysomes, and a corresponding increase of this mRNA in both monosomes and SGs, suggesting that these cells have a defect in translation-initiation that is specific for ALREX-containing mRNAs.

### RanBP2 Is Required for the Efficient Global Production of ER-Targeted Proteins

**Figure 8:**
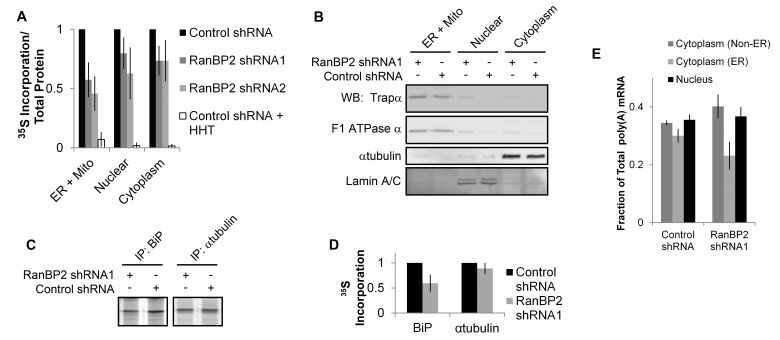
RanBP2 is required for the efficient translation of secretory proteins. (A–B) U2OS cells were treated with various lentiviruses, then 4 d post-infection cells were incubated in media containing [^35^S]-methionine/cysteine for 20 min. The cells were lysed and subfractionated by differential detergent treatments and centrifugation steps to produce three fractions: one enriched in nuclear proteins, a second containing ER and mitochondrial proteins, and the remaining cytoplasmic proteins. (A) The ratio of [^35^S]-incorporation to total protein from each fraction was measured and normalized to control cells. Each bar represents the average and standard error of three independent experiments. Note that radioactive incorporation into the fractions was blocked when cells were pre-treated with the translation inhibitor homoharingtonine (HHT) 15 min prior to [^35^S]-methionine/cysteine labeling, demonstrating that the fractions do not contain free (i.e., unincorporated) labeled amino acids. (B) Various cell fractions from knockdown and control cells were analyzed by immunoblot using antibodies against either Trapα (a resident ER protein), F1 ATPase α (a mitochondrial protein), α-tubulin (a cytoplasmic protein), and lamin A/C (a nuclear protein). (C–D) U2OS cells were treated with various lentiviruses, then 4 d post-infection cells were incubated in media containing [^35^S]-methionine/cysteine for 20 min. Cells were lysed and BiP and α-tubulin were immunoprecipitated with specific antibodies. The precipitates were then separated by SDS-PAGE and visualized by autoradiography. Band intensities were measured by densitometry, then normalized to control cells and the average and standard error between three independent experiments were plotted (D). (E) U2OS cells were treated with various lentiviruses, then 4 d post-infection cells were either immediately fixed to visualize cytoplasmic and nuclear poly(A) mRNA, or briefly permeabilized with digitonin to remove all cytoplasmic mRNA that was not anchored to the ER, and then fixed. Poly(A) mRNA was visualized by FISH staining with fluorescent oligo-dT probes and the fraction of fluorescence intensity associated with each compartment was calculated for at least 30 cells in a single experiment. Each bar represents the average and standard error between three separate experiments.

Previously we estimated that about 70% of all human genes that encode proteins targeted to either the ER or mitochondria, contain ALREX-promoting elements [7]. We thus decided to test the rate of protein synthesis in these various subcellular compartments. Newly synthesized proteins were labeled by feeding [^35^S]-methionine/cysteine to either control, or RanBP2-depleted cells. After 15 min, the cells were lysed, and the nuclear, ER/mitochondrial, and cytoplasmic fractions were isolated by differential centrifugation and analyzed for [^35^S] incorporation (Figure 8A) and protein composition by immunoblot (Figure 8B). In agreement with our previous bioinformatic analysis, the amount of newly synthesized protein in the ER/mitochondrial fraction decreased by half in cells depleted of RanBP2. We also detected a slight decrease in the amount of newly synthesized proteins in the nuclear and cytosolic fractions (Figure 8A), and this is likely due to the fact that a small but substantial fraction of non-secretory mRNAs contain features associated with ALREX (CC and FPR, unpublished observations). We also immunoprecipitated proteins from these cell lysates and determined that the amount of newly synthesized BiP, a lumenal ER chaperone that contains an adenine-depleted SSCR (see Table S1), significantly dropped in RanBP2 depleted cells (Figure 8C and 8D). In contrast the levels of α-tubulin remained unchanged. In agreement with our findings that RanBP2 is required for the translation of secretory mRNAs, we observed a small but statistically significant decrease in ER-associated mRNA after RanBP2 depletion (Figure 8E). As expected, the depletion of RanBP2 also generally promoted the general formation of SGs as detected by Tia1 staining; however, this was not seen in every experiment and the number of granules per cell was highly variable (unpublished data). In contrast, SGs were never detected in control cells.

In summary we conclude that RanBP2 is required for the efficient translation of mRNAs that encode secreted and membrane-bound proteins. Our data also suggest that cells depleted of RanBP2 accumulate mRNAs with defective translation initiation complexes, although the overall level of these defective complexes is likely very low and near the critical amount necessary to form SGs. In support of this, we did not detect a significant increase in ribosomal rRNA associated with the monosomal fraction after RanBP2 depletion (Figure 7A and 7B).

## Discussion

**Figure 9:**
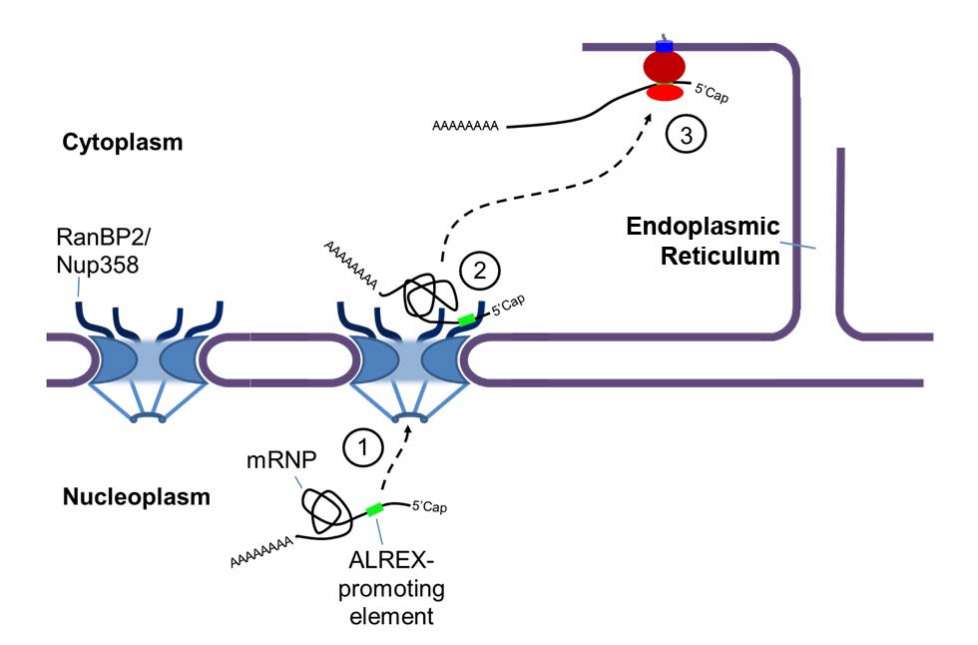
A general model of ALREX-regulated processes. (1) mRNAs containing ALREX-promoting SSCRs are packaged into mRNPs and exported by an unknown mechanism. (2) The exported mRNAs transiently interact with RanBP2 at the cytoplasmic face of the nuclear pore. This interaction likely causes some alteration within the mRNP. (3) Ultimately, this interaction promotes the translation of these mRNAs into secretory proteins at the surface of the ER.

In this study, we provide the first evidence that ALREX-promoting SSCRs act as a platform to coordinate both nuclear export and translation by recruiting factors to the 5′ end of the transcript. Our data suggest that once export is completed, this platform directly binds to the ZFRs of RanBP2, a large nucleoporin present on the cytoplasmic face of the nuclear pore. This interaction is required for downstream events, which likely includes the assembly of a competent translation initiation complex (Figure 9). Our data indicate that these events are critical for the proper translation of many mRNAs that encode secretory, and likely mitochondrial proteins. Our results may explain previous studies that reported that the presence of a signal sequence enhances protein synthesis (for example [46]), and that the translation rate for ER-bound transcripts was much greater than those found in the cytoplasm [47].

It is still unclear how ALREX-promoting elements enhance mRNA export through the pore before these mRNAs encounter RanBP2. Our data suggest that it is unlikely that the nuclear transport complex TAP/p15 binds directly to the element, as purified TAP/p15 heterodimers do not associate with either the *Ins* or *MHC* SSCRs in vitro (unpublished data). It is possible that other proteins that contain RanBP2-like zinc fingers can bind to the ALREX-promoting elements, as these are known to interact with adenine-poor motifs. For example, each of the two RanBP2-like zinc fingers of ZRanB2 interacts with a GGU triplet [48], and the RanBP2-like zinc finger of TLS recognizes GGUG [48],[49]. One obvious candidate ALREX-element binding protein is Nup153, whose zinc fingers share the highest degree of similarity to those of RanBP2. However, in our experiments we did not detect any association of this protein with the *Ins* SSCR in HeLa nuclear extracts (Figure 3F). In line with these results, the number of Nup153 ZFRs does not appear to correlate with adenine depletion across genomes (unpublished data). In particular, the *P. humanus* Nup153 ortholog has five zinc fingers, without any SSCR-specific adenine depletion. Identification of other proteins that associate with ALREX-promoting elements will be critical for our understanding of the molecular mechanisms that drive the ALREX pathway.

Since RanBP2 is thought mainly to reside on the cytoplasmic face of the nuclear pore, it is likely that this protein only interacts with mRNAs after they have been exported. This interaction may be required to remodel the messenger ribonucleoparticle (mRNP) complex after it has emerged from the nuclear pore. The idea is supported by recent observations of single mRNA molecules [50]. These studies demonstrated that after their nuclear export, a subset displayed extended dwell times in a region that was 100–150 nm away from the nuclear pore, a region that is occupied mainly by RanBP2 and presumably its paralogs. In addition to its ZFD, RanBP2 may have additional RNA-interacting domains [51], and thus also impact the translation of supplementary subclasses of mRNA. Furthermore, other factors that have been implicated in both nuclear export and translation, such as Dbp5 and Gle1, are also thought to be present on the cytoplasmic face of the nuclear pore [52],[53]. Thus it is likely that many critical mRNP remodeling events may occur in this region.

Since it is likely that the first translating ribosome would disrupt the association between the SSCR and RanBP2, it is likely that this protein must enhance the translatability of the mRNA before the pioneer round of translation [54]. This is reminiscent of the exon junction complex (EJC), which is known to stimulate the translatability of any associated mRNA and whose binding to the transcript is also displaced by translating ribosomes [55]. The EJC accomplishes this in part by promoting the phosphorylation of translation factors [56]. Recently, the EJC has been shown to associate with certain motifs that are found in the first exon [57], which resemble sequences that are enriched in ALREX-promoting SSCRs [7]. Experiments are currently underway to determine the exact contribution of the EJC to the translation of mRNAs containing ALREX-promoting SSCRs.

Although RanBP2 is not known to be associated with any kinases, it does have the ability to sumoylate proteins. Indeed a large proportion of known sumoylated proteins have RNA-binding activity [58]–[60]. Interestingly, it has been reported that the sumoylation of the cytoplasmic cap-binding protein, eIF4E, potentiates translation initiation [61]. Indeed, RanBP2 and eIF4E appear to cross regulate one another and thus impacting overall gene expression [62]. We have tried to recapitulate the potentiation of translation using in vitro translation systems, such as reticulocyte lysate; however, we could not detect any change between *MHC-ftz* and *2Ile-MHC-ftz* (Figure S1C). This is not surprising as these lysates lack RanBP2 (Figure S6). Unfortunately, adding HeLa nuclear extracts to reticulocyte lysates inhibits translation of all tested mRNAs (unpublished data). Experiments are now underway that aim to dissect the exact mechanism by which RanBP2 potentiates translation by performing a detailed domain analysis of this protein using an in vivo assay.

Finally it is possible that RanBP2 may play some role in delivering mRNAs to their ultimate destinations. Interestingly, in photoreceptor cells of the retina, it has been reported that RanBP2 can accompany vesicles that pinch off from the nuclear envelope to be delivered to the cell periphery, which contains both ER and mitochondria [63]. In other studies, RanBP2 has also been reported to interact with kinesins [64],[65] and dynein [65]. Further investigations will need to be performed to determine if RanBP2 plays a direct role in delivering secretory mRNAs to the ER.

## Materials and Methods

### Identification of ALREX-Binding Proteins

100 µl HeLa nuclear extract (7 mg/ml), which was prepared as previously described [66], was pre-cleared with streptavidin-coated magnetic beads (Dynabeads, Invitrogen), mixed with 10 µl denatured *E. coli* tRNA (20 mg/ml, Sigma), 10 µl salmon sperm DNA (11 mg/ml, Sigma), 5 µl of RNase-free BSA (20 mg/ml, Ambion), 250 µl of 2× binding buffer (0.1% TritonX-100, 1.2 M NaCl, 10 mM MgCl_2_, 2 mM DTT), and incubated with 20 µl of beads that were pre-bound with 10 µg of *Ins*, *7A-Ins* and βG biotinylated RNA (see the methods section in Text S1 for biotinylated RNA synthesis) for 1 h at 4°C with gentle rotation. The beads were then washed five times by incubation with 500 µl of 1× binding buffer (0.1% TritonX-100, 600 mM NaCl, 5 mM MgCl_2_, 1 mM DTT; Figure 3E). The beads were then isolated, treated with 10 µl of RNase solution (0.1% TritonX-100, 100 mM NaCl, 1 mg/ml RNase A, Sigma) for 15 min at room temperature. The supernatant was removed (Figure 3E, lanes 2–5) and the beads were mixed with 20 µl of 2× Laemmli sample buffer and incubated at 90°C for 5 min and separated by SDS-PAGE on a 4%–20% gradient gel. The gel was either silver stained (SilverQuest, Invitrogen) or transferred to nitrocellulose for immunoblotting (see the methods section in Text S1 for details). All silver-stained protein bands were cut and identified by microcapillary liquid chromatography tandem mass spectrometry (Taplin Mass Spectrometry Facility, Harvard Medical School).

### Expression and Purification of RanBP2 Fragments and Ran

The RanBP2 TPR domain (amino acid residues 1–601), RBR1 (residues 514–1,245), ZFD (residues 1,335–1,829), RBR2 (residues 1,832–2,553), C-Term (residues 2,765–3,138) were amplified from the pBSK-RanBP2 [28] and cloned into pET28a vector (Novagen) using restriction-free cloning with the addition of N-terminal His-tag.

### Electromobility Shift Assays

[^32^P]-labeled RNA was incubated with Hela cell nuclear extract (0.44 µg/µl final protein concentration), RanBP2 fragments, or BSA (120 ng/µl) in 1.5× φ buffer (1× φ buffer: 150 mM KAcetate, 5 mM MgAcetate, 20 mM HEPES [pH 7.4]) with 10 µg/ml denatured yeast tRNA at room temperature for 15 min. For the competition EMSA experiments, unlabeled RNA was first mixed with tRNA and radiolabeled RNA, then incubated with nuclear extract or recombinant proteins. For the Ran competition assay, 170 ng/µl BSA or GST-Ran was pre-incubated with 50 ng/µl RanBP2 ZFD, then incubated with labeled RNA. RNA-protein complexes were resolved by native PAGE (TBE, 3.5%, 5%, and 10% acrylamide; acrylamide/bisacrylamide ratio of 19∶1). Radiolabelled RNA was visualized using a Typhoon phosphoimager (GE Healthcare).

### Cell Biological Methods

Cell culture, transfection, microinjection, extraction, fixation, FISH, and immunofluorescence were performed as previously described [6],[13],[67]. For more information see the methods section in Text S1.

## Supporting Information

Figure S1 **The translational products of the** ***MHC-ftz*** **and** ***2Ile-MHC-ftz*** **mRNAs are translocated into the ER where they are processed into identical protein products.** (A–B) Lysates were collected from U2OS cells expressing MHC-ftz, 2Ile-MHC-ftz, or a version of this protein translated from a mutant frame-shifted *MHC-ftz* (*FS-MHC-ftz*). This last construct contains a point insertion before, and a point deletion after the SSCR, thus encoding a cytoplasmic version of the ftz protein (see [6] and Table S1). The lysates were first treated with Endo H, which removes ER-specific N-linked glycosylation, or PNGase, which removes all N-linked glycosylation. The lysates were then denatured and separated by SDS-PAGE, and probed with antibodies against the HA epitope (A–B), and against α-tubulin as a loading control (A). An image of colored molecular weight standards is included in (A). Note that after either Endo H or PNGase treatment, the mobility of all of the MHC-ftz and 2Ile-MHC-ftz proteins increased. This is to be expected as the mature ftz polypeptide has two consensus glycosylation sites. In contrast FS-MHC-ftz protein was unaffected by PNGase treatment. From these data we concluded that MHC-ftz, but not FS-MHC-ftz, is present in the ER lumen where it is glycosylated. Also note that the mobility of deglycosylated MHC-ftz is greater than that of FS-MHC-ftz despite the fact that the two protein products should have similar molecular weights, suggesting that the former is processed by signal peptidase. (C) In vitro transcribed and capped mRNAs were translated in vitro using reticulocyte lysate in the presence of [^35^S]-methionine/cysteine. Samples were separated by SDS-PAGE, and newly synthesized proteins were detected by autoradiography. An image of colored molecular weight markers (MWMs) was included as a reference. Note that reticulocyte lysate does not contain ER-derived microsomes, thus the resulting ftz protein products are expected to be unprocessed and unmodified. Also note that the molecular weight of these in vitro synthesized proteins (>17 kD) was greater than the final deglycosylated in vivo translated forms (see PNGase and Endo H treated samples in (A), which are <17 kD). This observation indicates that in cells, MHC-ftz and 2Ile-MHC-ftz are proteolytically processed, likely by signal peptidase. (D–F) To calculate the half-life of the two protein products, U2OS cells that were transfected with plasmids that contained either the *MHC-ftz* or *2Ile-MHC-ftz* genes (18–24 h post-transfection) were treated with cyclohexamide (CHX) for indicated time points. Cell lysates were collected and separated by SDS-PAGE, then probed with antibodies against the HA epitope and α-tubulin. (D) The amount of ftz protein (*y*-axis) at each time point (*x*-axis) was analyzed by densitometry and then normalized to the initial protein level and plotted. Each data point represents the average and standard deviation of three independent experiments. (F) Each bar represents the average and standard error of the half-lives derived from three independent experiments.
(TIF)

Figure S2 **Identification of ALREX-element associating proteins from HeLa nuclear extract.** Streptavidin-coated magnetic beads, bound with various biotinylated RNAs (each 76 nucleotides long) or without any RNA (“No RNA”), were used to isolate ALREX-element associating proteins from NE under low salt conditions (i.e., 200 mM NaCl instead of the 600 mM used in Figure 3E–3G). Beads were treated with RNase A and then proteins were denatured in SDS at 90°C for 5 min. Eluted proteins were separated on a 4%–20% gradient gel and silver stained. ALREX-interacting proteins (RanBP2, Importinβ, RanGAP1, and Ran) are indicated. Note that under these conditions, many non-specific proteins are present in the RNA-coated beads at similar levels, but not in the uncoated beads. These observations indicate that the RNA-coated beads contained similar amounts of bound oligonucleotides. The position of molecular weight markers in kDa are indicated on the right.
(TIF)

Figure S3 **RanBP2/Nup358 is required for ALREX-mediated translation enhancement.** (A–C) U2OS cells treated with shRNA1 against RanBP2 or control lentiviruses. Three days after infection the cells were transfected with plasmids containing various *CALR-HA* constructs and *H1B-GFP*. The level of protein and mRNA was analyzed by densitometry analysis of HA and GFP immunoblots (A–B), and *ftz* and *GFP* northern blot (C). Each bar represents the average level and the standard error between five independent experiments. (D) U2OS cells treated with either shRNA2 against RanBP2 or control virus. Four days post-infection the cell lysates were collected and analyzed by immunoblot for RanBP2 and α-tubulin. (E–J) U2OS cells treated with either shRNA1 or shRNA2 against RanBP2 or control virus, were transfected with various *ftz* (E–F, I–J) or *CALR-HA* (G–H) constructs along with *H1B-GFP*. Protein and mRNA levels were then analyzed by densitometry analysis of immunoblots (E, G, I) and northern blots (F, H, J). Each bar represents the average level and the standard error of protein (E, G) or mRNA (F, H) levels between three independent experiments.
(TIF)

Figure S4 **Level of co-expression of GFP-RanBP2 and MHC-ftz constructs in control and knockdown U2OS cells.** Cells were treated with lentiviruses that delivered RanBP2 shRNA3 or control shRNA. Three days post-infection cells were co-transfected with plasmids containing MHC-ftz and either GFP-RanBP2 (A) or GFP-RanBP2Δ6ZFR (B). 48 h post-transfection cells were fixed and immunostained for HA and GFP using specific antibodies. Cells expressing detectable levels of either protein alone or together were tabulated. For each experiment, the expression of at least 400 cells was tabulated.
(TIF)

Figure S5 **Densitometry analysis of ribosome intensities in sucrose gradients.** For each polysome gradient, ribosomal rRNA was visualized as in Figure 7A and 7B and the intensity of the 28S rRNA and 18S rRNA bands were calculated by densitometry analysis. The fraction of the ribosomal rRNAs in each sucrose gradient fraction was then averaged between experiments. Each data point represents the average and standard error between three independent experiments.
(TIF)

Figure S6 **Rabbit reticulocyte lysate does not contain RanBP2/Nup358.** Rabbit reticulocyte lysates, and lysates from U2OS cells treated with shRNA1 against RanBP2 or control lentiviruses for 4 d, were separated by SDS-PAGE and immunoblotted for RanBP2 and α-tubulin.
(TIF)

Table S1 **Constructs used in this study.** Nucleotides and encoded amino acid sequences for all ftz and CALR constructs used in this study. Also included are the nucleotides and encoded amino acid sequences of human BiP. Note that all nucleotide and amino acid substitutions in 2Ile-MHC, 2Ile-INS, 7A-INS, and 4Ile-CALR are in bold.
(XLS)

Table S2 **Analysis of SSCR-specific adenine depletion in metazoans.** The mean and standard error of the mean of the longest A-less tract in the first 99 nucleotides in ORFs encoding signal sequences. As a control a random 99 nucleotides from each ORF was analyzed. Also listed are the number of ZFRs in that organism's RanBP2 ortholog, and the total number of ORFs analyzed.
(XLS)

Text S1 **Additional supplemental materials and methods.**
(DOC)

## Abbreviations

ALREX: alternative mRNA nuclear export
*βG*: *β-globin*
CALR: calreticulin
EJC: exon junction complex
EMSA: electro mobility shift assay
ER: endoplasmic reticulum
FISH: fluorescence in situ hybridization
ftz: *fushi tarazu*
GFP: green fluorescent protein
H1B: histone 1B
INS or Ins: insulin
MHC: major histocompatibility complex
mRNP: messenger ribonucleoparticle
PRP: prion protein
PTH: parathyroid hormone
SG: stress granule
SSCR: signal sequence coding region
ZFD: zinc finger domain
ZFR: zinc finger repeat

## References

[1] Moore MJ, Proudfoot NJ (2009) Pre-mRNA processing reaches back to transcription and ahead to translation. Cell 136: 688–700. 19239889 10.1016/j.cell.2009.02.001

[2] Palazzo AF, Akef A (2012) Nuclear export as a key arbiter of “mRNA identity” in eukaryotes. Biochim Biophys Acta 1819: 566–577. 22248619 10.1016/j.bbagrm.2011.12.012

[3] Cheng H, Dufu K, Lee C-S, Hsu JL, Dias A, et al (2006) Human mRNA export machinery recruited to the 5′ end of mRNA. Cell 127: 1389–1400. 17190602 10.1016/j.cell.2006.10.044

[4] Strässer K, Hurt E (2000) Yra1p, a conserved nuclear RNA-binding protein, interacts directly with Mex67p and is required for mRNA export. EMBO J 19: 410–420. 10722314 10.1093/emboj/19.3.410 PMC305578

[5] Stutz F, Bachi A, Doerks T, Braun IC, Séraphin B, et al (2000) REF, an evolutionary conserved family of hnRNP-like proteins, interacts with TAP/Mex67p and participates in mRNA nuclear export. RNA 6: 638–650. 10786854 10.1017/s1355838200000078 PMC1369944

[6] Palazzo AF, Springer M, Shibata Y, Lee C-S, Dias AP, et al (2007) The signal sequence coding region promotes nuclear export of mRNA. PLoS Biol 5: e322 doi:10.1371/journal.pbio.0050322. 18052610 10.1371/journal.pbio.0050322 PMC2100149

[7] Cenik C, Chua HN, Zhang H, Tarnawsky S, Akef A, et al (2011) Genome analysis reveals interplay between 5′UTR introns and nuclear mRNA export for secretory and mitochondrial genes. PLoS Genet 7: e1001366 doi:10.1371/journal.pgen.1001366. 21533221 10.1371/journal.pgen.1001366 PMC3077370

[8] Tarnawsky SP, Palazzo AF (2012) Positional requirements for the stimulation of mRNA nuclear export by ALREX-promoting elements. Mol Biosyst 8: 2527–2530. 22373716 10.1039/c2mb25016k

[9] Mazroui R, Sukarieh R, Bordeleau M-E, Kaufman RJ, Northcote P, et al (2006) Inhibition of ribosome recruitment induces stress granule formation independently of eukaryotic initiation factor 2alpha phosphorylation. Mol Biol Cell 17: 4212–4219. 16870703 10.1091/mbc.E06-04-0318 PMC1635342

[10] Mokas S, Mills JR, Garreau C, Fournier M-J, Robert F, et al (2009) Uncoupling stress granule assembly and translation initiation inhibition. Mol Biol Cell 20: 2673–2683. 19369421 10.1091/mbc.E08-10-1061 PMC2688547

[11] Reed R, Maniatis T (1985) Intron sequences involved in lariat formation during pre-mRNA splicing. Cell 41: 95–105. 3888410 10.1016/0092-8674(85)90064-9

[12] Luo MJ, Reed R (1999) Splicing is required for rapid and efficient mRNA export in metazoans. Proc Natl Acad Sci U S A 96: 14937–14942. 10611316 10.1073/pnas.96.26.14937 PMC24751

[13] Gueroussov S, Tarnawsky SP, Cui XA, Mahadevan K, Palazzo AF (2010) Analysis of mRNA nuclear export kinetics in mammalian cells by microinjection. J Vis Exp 46: 2387. 10.3791/2387 PMC3159668 21178962

[14] Ikemura T (1985) Codon usage and tRNA content in unicellular and multicellular organisms. Mol Biol Evol 2: 13–34. 3916708 10.1093/oxfordjournals.molbev.a040335

[15] Duret L (2000) tRNA gene number and codon usage in the C. elegans genome are co-adapted for optimal translation of highly expressed genes. Trends Genet 16: 287–289. 10858656 10.1016/s0168-9525(00)02041-2

[16] Novoa EM, Pavon-Eternod M, Pan T, Ribas de Pouplana L (2012) A role for tRNA modifications in genome structure and codon usage. Cell 149: 202–213. 22464330 10.1016/j.cell.2012.01.050

[17] Kendall DA, Kaiser ET (1988) A functional decaisoleucine-containing signal sequence. Construction by cassette mutagenesis. J Biol Chem 263: 7261–7265. 3284884

[18] Kaiser CA, Botstein D (1990) Efficiency and diversity of protein localization by random signal sequences. Mol Cell Biol 10: 3163–3173. 2160595 10.1128/mcb.10.6.3163-3173.1990 PMC360681

[19] Hessa T, Kim H, Bihlmaier K, Lundin C, Boekel J, et al (2005) Recognition of transmembrane helices by the endoplasmic reticulum translocon. Nature 433: 377–381. 15674282 10.1038/nature03216

[20] Nakamura Y, Gojobori T, Ikemura T (2000) Codon usage tabulated from international DNA sequence databases: status for the year 2000. Nucleic Acids Res 28: 292. 10592250 10.1093/nar/28.1.292 PMC102460

[21] Hurt JA, Obar RA, Zhai B, Farny NG, Gygi SP, et al (2009) A conserved CCCH-type zinc finger protein regulates mRNA nuclear adenylation and export. J Cell Biol 185: 265–277. 19364924 10.1083/jcb.200811072 PMC2700372

[22] Jensen TH, Patricio K, McCarthy T, Rosbash M (2001) A block to mRNA nuclear export in S. cerevisiae leads to hyperadenylation of transcripts that accumulate at the site of transcription. Mol Cell 7: 887–898. 11336711 10.1016/s1097-2765(01)00232-5

[23] Williams DB (2006) Beyond lectins: the calnexin/calreticulin chaperone system of the endoplasmic reticulum. J Cell Sci 119: 615–623. 16467570 10.1242/jcs.02856

[24] Kedersha NL, Gupta M, Li W, Miller I, Anderson P (1999) RNA-binding proteins TIA-1 and TIAR link the phosphorylation of eIF-2 alpha to the assembly of mammalian stress granules. J Cell Biol 147: 1431–1442. 10613902 10.1083/jcb.147.7.1431 PMC2174242

[25] Kedersha N, Chen S, Gilks N, Li W, Miller IJ, et al (2002) Evidence that ternary complex (eIF2-GTP-tRNA(i)(Met))-deficient preinitiation complexes are core constituents of mammalian stress granules. Mol Biol Cell 13: 195–210. 11809833 10.1091/mbc.01-05-0221 PMC65082

[26] Anderson P, Kedersha N (2006) RNA granules. J Cell Biol 172: 803–808. 16520386 10.1083/jcb.200512082 PMC2063724

[27] Valencia P, Dias AP, Reed R (2008) Splicing promotes rapid and efficient mRNA export in mammalian cells. Proc Natl Acad Sci U S A 105: 3386–3391. 18287003 10.1073/pnas.0800250105 PMC2265164

[28] Pichler A, Gast A, Seeler JS, Dejean A, Melchior F (2002) The nucleoporin RanBP2 has SUMO1 E3 ligase activity. Cell 108: 109–120. 11792325 10.1016/s0092-8674(01)00633-x

[29] Mahajan R, Delphin C, Guan T, Gerace L, Melchior F (1997) A small ubiquitin-related polypeptide involved in targeting RanGAP1 to nuclear pore complex protein RanBP2. Cell 88: 97–107. 9019411 10.1016/s0092-8674(00)81862-0

[30] Saitoh H, Pu R, Cavenagh M, Dasso M (1997) RanBP2 associates with Ubc9p and a modified form of RanGAP1. Proc Natl Acad Sci U S A 94: 3736–3741. 9108047 10.1073/pnas.94.8.3736 PMC20510

[31] Yokoyama N, Hayashi N, Seki T, Panté N, Ohba T, et al (1995) A giant nucleopore protein that binds Ran/TC4. Nature 376: 184–188. 7603572 10.1038/376184a0

[32] Wu J, Matunis MJ, Kraemer D, Blobel G, Coutavas E (1995) Nup358, a cytoplasmically exposed nucleoporin with peptide repeats, Ran-GTP binding sites, zinc fingers, a cyclophilin A homologous domain, and a leucine-rich region. J Biol Chem 270: 14209–14213. 7775481 10.1074/jbc.270.23.14209

[33] Forler D, Rabut G, Ciccarelli FD, Herold A, Köcher T, et al (2004) RanBP2/Nup358 provides a major binding site for NXF1-p15 dimers at the nuclear pore complex and functions in nuclear mRNA export. Mol Cell Biol 24: 1155–1167. 14729961 10.1128/MCB.24.3.1155-1167.2004 PMC321439

[34] Lévesque L, Bor Y-C, Matzat LH, Jin L, Berberoglu S, et al (2006) Mutations in tap uncouple RNA export activity from translocation through the nuclear pore complex. Mol Biol Cell 17: 931–943. 16314397 10.1091/mbc.E04-07-0634 PMC1356601

[35] Ben-Efraim I, Gerace L (2001) Gradient of increasing affinity of importin beta for nucleoporins along the pathway of nuclear import. J Cell Biol 152: 411–417. 11266456 10.1083/jcb.152.2.411 PMC2199621

[36] Davis LI, Blobel G (1986) Identification and characterization of a nuclear pore complex protein. Cell 45: 699–709. 3518946 10.1016/0092-8674(86)90784-1

[37] Davis LI, Blobel G (1987) Nuclear pore complex contains a family of glycoproteins that includes p62: glycosylation through a previously unidentified cellular pathway. Proc Natl Acad Sci U S A 84: 7552–7556. 3313397 10.1073/pnas.84.21.7552 PMC299337

[38] Loughlin FE, Mansfield RE, Vaz PM, McGrath AP, Setiyaputra S, et al (2009) The zinc fingers of the SR-like protein ZRANB2 are single-stranded RNA-binding domains that recognize 5′ splice site-like sequences. Proc Natl Acad Sci U S A 106: 5581–5586. 19304800 10.1073/pnas.0802466106 PMC2667063

[39] Higa MM, Alam SL, Sundquist WI, Ullman KS (2007) Molecular characterization of the Ran-binding zinc finger domain of Nup153. J Biol Chem 282: 17090–17100. 17426026 10.1074/jbc.M702715200

[40] Hamada M, Haeger A, Jeganathan KB, Van Ree JH, Malureanu L, et al (2011) Ran-dependent docking of importin-beta to RanBP2/Nup358 filaments is essential for protein import and cell viability. J Cell Biol 194: 597–612. 21859863 10.1083/jcb.201102018 PMC3160583

[41] Hutten S, Flotho A, Melchior F, Kehlenbach RH (2008) The Nup358-RanGAP complex is required for efficient importin alpha/beta-dependent nuclear import. Mol Biol Cell 19: 2300–2310. 18305100 10.1091/mbc.E07-12-1279 PMC2366868

[42] Kapadia F, Pryor A, Chang T-H, Johnson LF (2006) Nuclear localization of poly(A)+ mRNA following siRNA reduction of expression of the mammalian RNA helicases UAP56 and URH49. Gene 384: 37–44. 16949217 10.1016/j.gene.2006.07.010

[43] Ciccarelli FD, Von Mering C, Suyama M, Harrington ED, Izaurralde E, et al (2005) Complex genomic rearrangements lead to novel primate gene function. Genome Res 15: 343–351. 15710750 10.1101/gr.3266405 PMC551560

[44] Cui XA, Zhang H, Palazzo AF (2012) p180 promotes the ribosome-independent localization of a subset of mRNA to the endoplasmic reticulum. PLoS Biol 10: e1001336 doi:10.1371/journal.pbio.1001336. 22679391 10.1371/journal.pbio.1001336 PMC3362647

[45] Lerner RS, Seiser RM, Zheng T, Lager PJ, Reedy MC, et al (2003) Partitioning and translation of mRNAs encoding soluble proteins on membrane-bound ribosomes. RNA 9: 1123–1137. 12923260 10.1261/rna.5610403 PMC1370476

[46] Wicksteed B, Herbert TP, Alarcon C, Lingohr MK, Moss LG, et al (2001) Cooperativity between the preproinsulin mRNA untranslated regions is necessary for glucose-stimulated translation. J Biol Chem 276: 22553–22558. 11297542 10.1074/jbc.M011214200

[47] Stephens SB, Nicchitta CV (2008) Divergent regulation of protein synthesis in the cytosol and endoplasmic reticulum compartments of mammalian cells. Mol Biol Cell 19: 623–632. 18077556 10.1091/mbc.E07-07-0677 PMC2230589

[48] Nguyen CD, Mansfield RE, Leung W, Vaz PM, Loughlin FE, et al (2011) Characterization of a family of RanBP2-type zinc fingers that can recognize single-stranded RNA. J Mol Biol 407: 273–283. 21256132 10.1016/j.jmb.2010.12.041

[49] Lerga A, Hallier M, Delva L, Orvain C, Gallais I, et al (2001) Identification of an RNA binding specificity for the potential splicing factor TLS. J Biol Chem 276: 6807–6816. 11098054 10.1074/jbc.M008304200

[50] Grünwald D, Singer RH (2010) In vivo imaging of labelled endogenous β-actin mRNA during nucleocytoplasmic transport. Nature 467: 604–607. 20844488 10.1038/nature09438 PMC3005609

[51] Kassube SA, Stuwe T, Lin DH, Antonuk CD, Napetschnig J, et al (2012) Crystal structure of the N-terminal domain of Nup358/RanBP2. J Mol Biol 423: 752–765. 22959972 10.1016/j.jmb.2012.08.026 PMC4226657

[52] Bolger TA, Folkmann AW, Tran EJ, Wente SR (2008) The mRNA export factor Gle1 and inositol hexakisphosphate regulate distinct stages of translation. Cell 134: 624–633. 18724935 10.1016/j.cell.2008.06.027 PMC2601711

[53] Gross T, Siepmann A, Sturm D, Windgassen M, Scarcelli JJ, et al (2007) The DEAD-box RNA helicase Dbp5 functions in translation termination. Science 315: 646–649. 17272721 10.1126/science.1134641

[54] Maquat LE, Tarn W-Y, Isken O (2010) The pioneer round of translation: features and functions. Cell 142: 368–374. 20691898 10.1016/j.cell.2010.07.022 PMC2950652

[55] Nott A, Le Hir H, Moore MJ (2004) Splicing enhances translation in mammalian cells: an additional function of the exon junction complex. Genes Dev 18: 210–222. 14752011 10.1101/gad.1163204 PMC324426

[56] Ma XM, Yoon S-O, Richardson CJ, Jülich K, Blenis J (2008) SKAR links pre-mRNA splicing to mTOR/S6K1-mediated enhanced translation efficiency of spliced mRNAs. Cell 133: 303–313. 18423201 10.1016/j.cell.2008.02.031

[57] Singh G, Kucukural A, Cenik C, Leszyk JD, Shaffer SA, et al (2012) The cellular EJC interactome reveals higher-order mRNP structure and an EJC-SR protein nexus. Cell 151: 750–764. 23084401 10.1016/j.cell.2012.10.007 PMC3522173

[58] Nie M, Xie Y, Loo JA, Courey AJ (2009) Genetic and proteomic evidence for roles of Drosophila SUMO in cell cycle control, Ras signaling, and early pattern formation. PLoS ONE 4: e5905 doi:10.1371/journal.pone.0005905. 19529778 10.1371/journal.pone.0005905 PMC2692000

[59] Makhnevych T, Sydorskyy Y, Xin X, Srikumar T, Vizeacoumar FJ, et al (2009) Global map of SUMO function revealed by protein-protein interaction and genetic networks. Mol Cell 33: 124–135. 19150434 10.1016/j.molcel.2008.12.025

[60] Meier I (2012) mRNA export and sumoylation-Lessons from plants. Biochim Biophys Acta 1819: 531–537. 22306659 10.1016/j.bbagrm.2012.01.006

[61] Xu X, Vatsyayan J, Gao C, Bakkenist CJ, Hu J (2010) Sumoylation of eIF4E activates mRNA translation. EMBO Rep 11: 299–304. 20224576 10.1038/embor.2010.18 PMC2854592

[62] Culjkovic-Kraljacic B, Baguet A, Volpon L, Amri A, Borden KLB (2012) The oncogene eIF4E reprograms the nuclear pore complex to promote mRNA export and oncogenic transformation. Cell Rep 2: 207–215. 22902403 10.1016/j.celrep.2012.07.007 PMC3463940

[63] Mavlyutov TA, Cai Y, Ferreira PA (2002) Identification of RanBP2- and kinesin-mediated transport pathways with restricted neuronal and subcellular localization. Traffic 3: 630–640. 12191015 10.1034/j.1600-0854.2002.30905.x

[64] Cai Y, Singh BB, Aslanukov A, Zhao H, Ferreira PA (2001) The docking of kinesins, KIF5B and KIF5C, to Ran-binding protein 2 (RanBP2) is mediated via a novel RanBP2 domain. J Biol Chem 276: 41594–41602. 11553612 10.1074/jbc.M104514200

[65] Murawala P, Tripathi MM, Vyas P, Salunke A, Joseph J (2009) Nup358 interacts with APC and plays a role in cell polarization. J Cell Sci 122: 3113–3122. 19654215 10.1242/jcs.037523

[66] Mayeda A, Krainer AR (1999) Preparation of HeLa cell nuclear and cytosolic S100 extracts for in vitro splicing. Methods Mol Biol 118: 309–314. 10549533 10.1385/1-59259-676-2:309

[67] Cui XA, Palazzo AF (2012) Visualization of Endoplasmic Reticulum Localized mRNAs in Mammalian Cells. J Vis Exp 70: e50066. 10.3791/50066 PMC3575208 23271194

