# Supplementary figures and images for: RanBP2/Nup358 Potentiates the Translation of a Subset of mRNAs Encoding Secretory Proteins
Source: PLoS Biol. 2013 Apr 23;11(4):e1001545. doi: 10.1371/journal.pbio.1001545 (PMC3635865; doi:10.1371/journal.pbio.1001545)

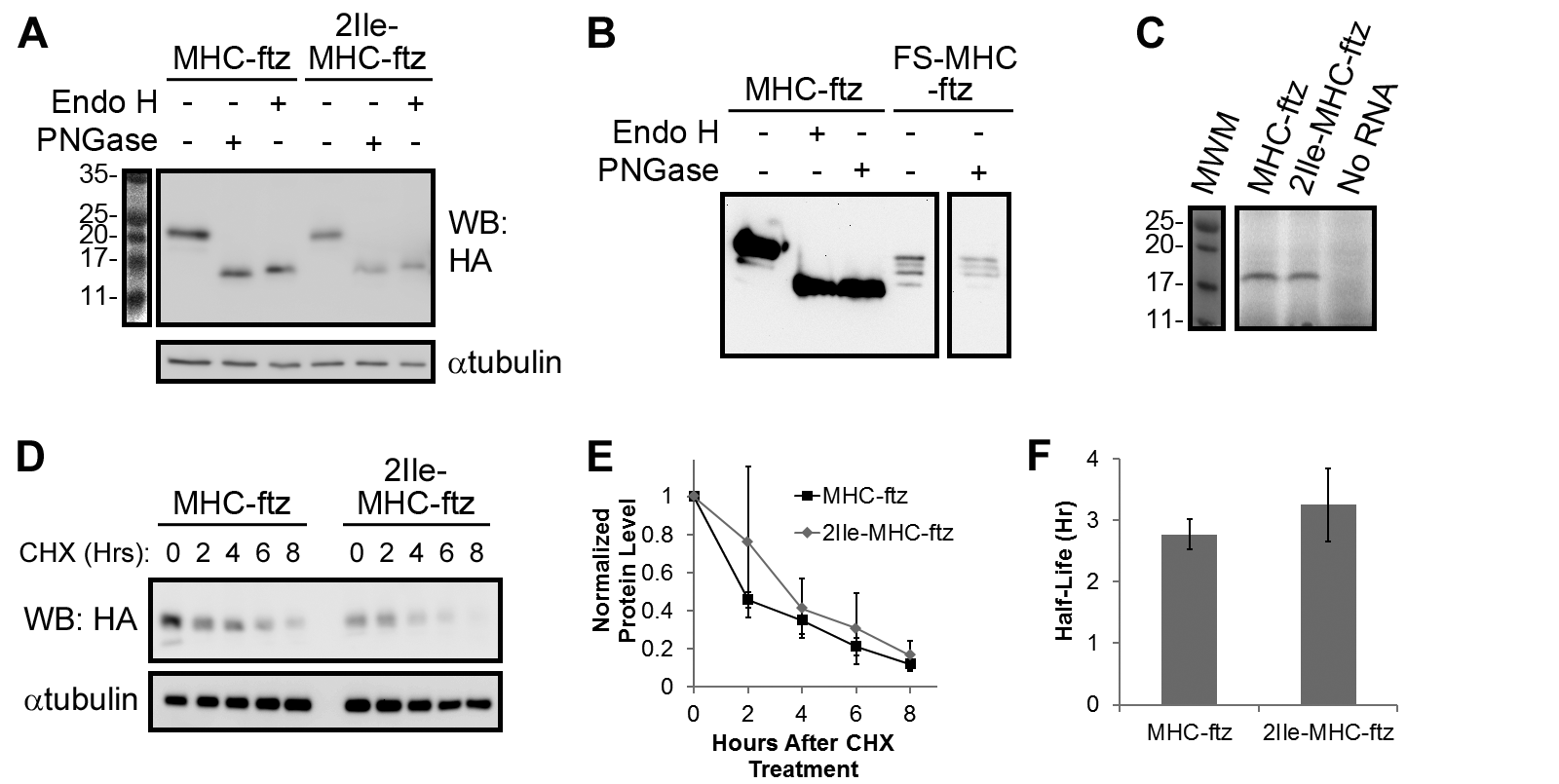

Supplement: Figure S1 — The translational products of the MHC-ftz and 2Ile-MHC-ftz mRNAs are translocated into the ER where they are processed into identical protein products. (A–B) Lysates were collected from U2OS cells expressing MHC-ftz, 2Ile-MHC-ftz, or a version of this protein translated from a mutant frame-shifted MHC-ftz (FS-MHC-ftz). This last construct contains a point insertion before, and a point deletion after the SSCR, thus encoding a cytoplasmic version of the ftz protein (see [6] and Table S1). The lysates were first treated with Endo H, which removes ER-specific N-linked glycosylation, or PNGase, which removes all N-linked glycosylation. The lysates were then denatured and separated by SDS-PAGE, and probed with antibodies against the HA epitope (A–B), and against α-tubulin as a loading control (A). An image of colored molecular weight standards is included in (A). Note that after either Endo H or PNGase treatment, the mobility of all of the MHC-ftz and 2Ile-MHC-ftz proteins increased. This is to be expected as the mature ftz polypeptide has two consensus glycosylation sites. In contrast FS-MHC-ftz protein was unaffected by PNGase treatment. From these data we concluded that MHC-ftz, but not FS-MHC-ftz, is present in the ER lumen where it is glycosylated. Also note that the mobility of deglycosylated MHC-ftz is greater than that of FS-MHC-ftz despite the fact that the two protein products should have similar molecular weights, suggesting that the former is processed by signal peptidase. (C) In vitro transcribed and capped mRNAs were translated in vitro using reticulocyte lysate in the presence of [35S]-methionine/cysteine. Samples were separated by SDS-PAGE, and newly synthesized proteins were detected by autoradiography. An image of colored molecular weight markers (MWMs) was included as a reference. Note that reticulocyte lysate does not contain ER-derived microsomes, thus the resulting ftz protein products are expected to be unprocessed and unmodified. Also note that the [file pbio.1001545.s001.tif]

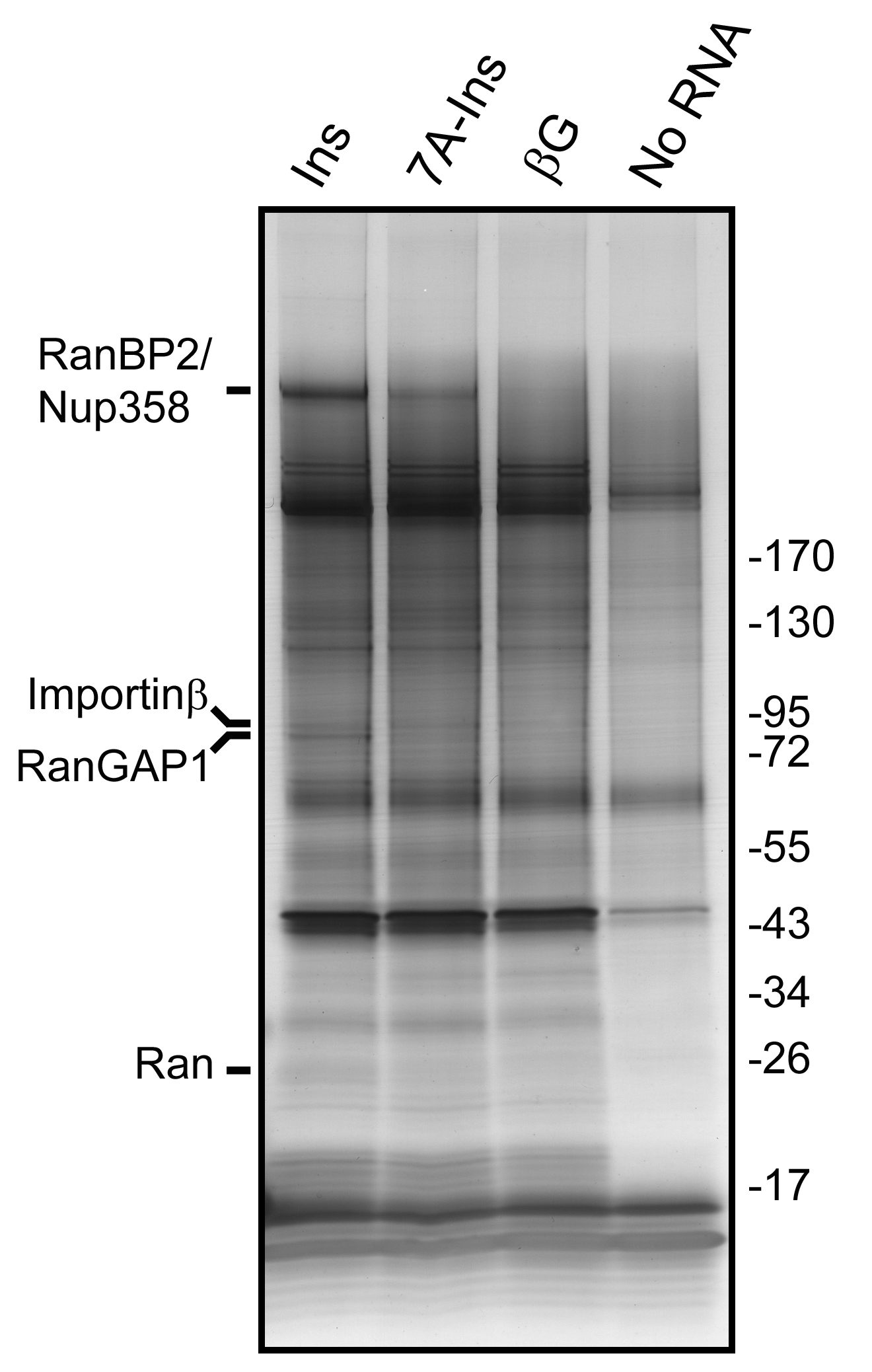

Supplement: Figure S2 — Identification of ALREX-element associating proteins from HeLa nuclear extract. Streptavidin-coated magnetic beads, bound with various biotinylated RNAs (each 76 nucleotides long) or without any RNA (“No RNA”), were used to isolate ALREX-element associating proteins from NE under low salt conditions (i.e., 200 mM NaCl instead of the 600 mM used in Figure 3E–3G). Beads were treated with RNase A and then proteins were denatured in SDS at 90°C for 5 min. Eluted proteins were separated on a 4%–20% gradient gel and silver stained. ALREX-interacting proteins (RanBP2, Importinβ, RanGAP1, and Ran) are indicated. Note that under these conditions, many non-specific proteins are present in the RNA-coated beads at similar levels, but not in the uncoated beads. These observations indicate that the RNA-coated beads contained similar amounts of bound oligonucleotides. The position of molecular weight markers in kDa are indicated on the right. (TIF) [file pbio.1001545.s002.tif]

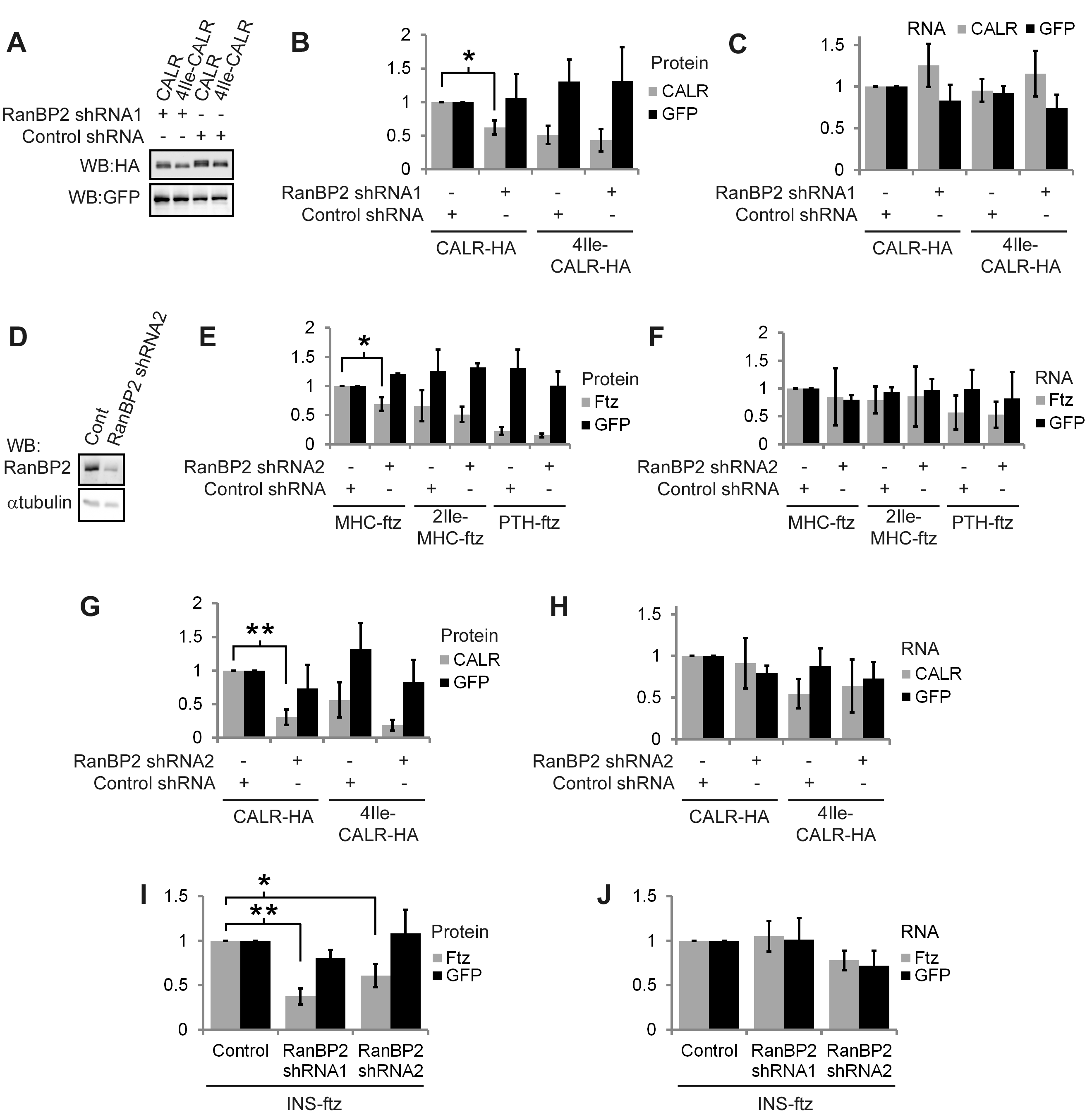

Supplement: Figure S3 — RanBP2/Nup358 is required for ALREX-mediated translation enhancement. (A–C) U2OS cells treated with shRNA1 against RanBP2 or control lentiviruses. Three days after infection the cells were transfected with plasmids containing various CALR-HA constructs and H1B-GFP. The level of protein and mRNA was analyzed by densitometry analysis of HA and GFP immunoblots (A–B), and ftz and GFP northern blot (C). Each bar represents the average level and the standard error between five independent experiments. (D) U2OS cells treated with either shRNA2 against RanBP2 or control virus. Four days post-infection the cell lysates were collected and analyzed by immunoblot for RanBP2 and α-tubulin. (E–J) U2OS cells treated with either shRNA1 or shRNA2 against RanBP2 or control virus, were transfected with various ftz (E–F, I–J) or CALR-HA (G–H) constructs along with H1B-GFP. Protein and mRNA levels were then analyzed by densitometry analysis of immunoblots (E, G, I) and northern blots (F, H, J). Each bar represents the average level and the standard error of protein (E, G) or mRNA (F, H) levels between three independent experiments. (TIF) [file pbio.1001545.s003.tif]

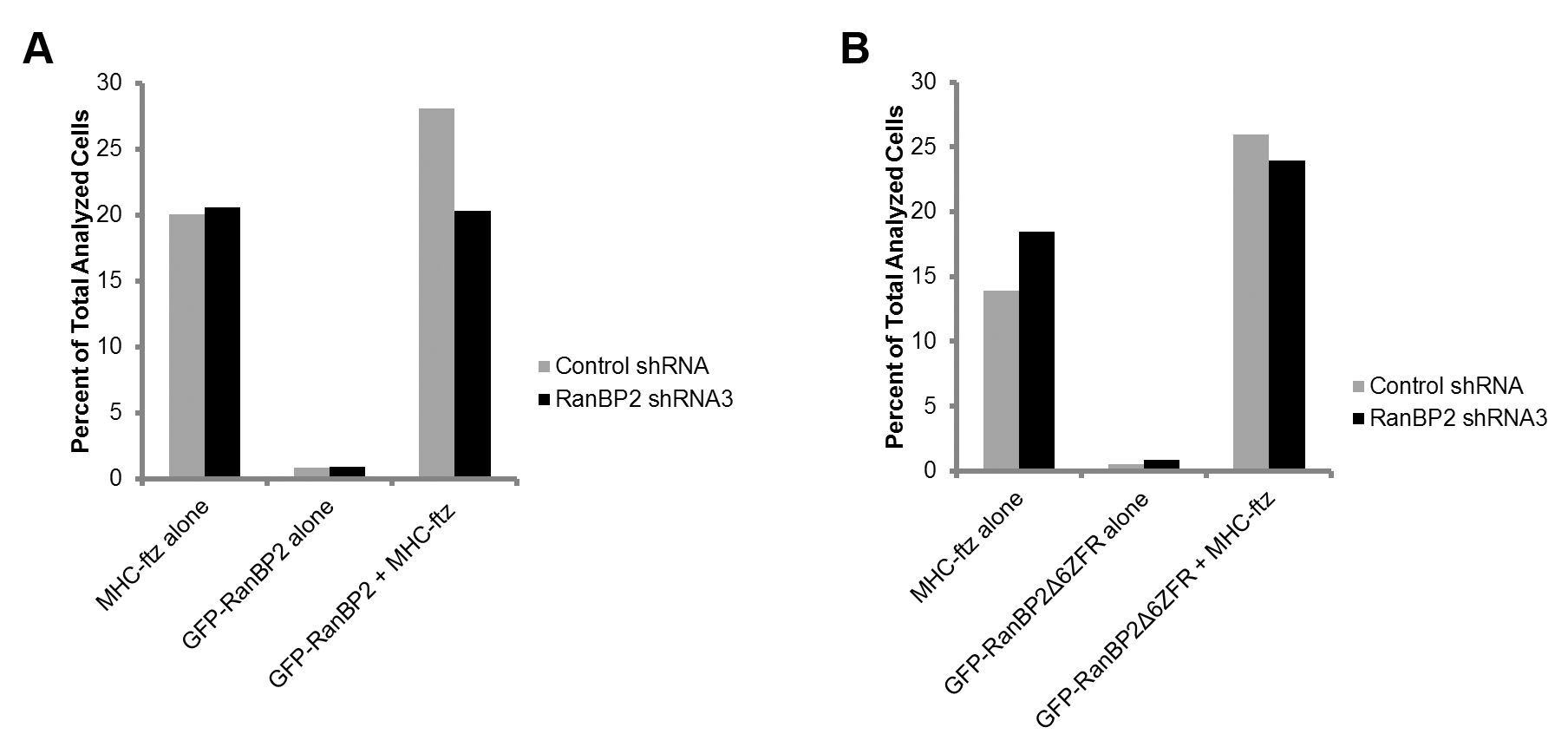

Supplement: Figure S4 — Level of co-expression of GFP-RanBP2 and MHC-ftz constructs in control and knockdown U2OS cells. Cells were treated with lentiviruses that delivered RanBP2 shRNA3 or control shRNA. Three days post-infection cells were co-transfected with plasmids containing MHC-ftz and either GFP-RanBP2 (A) or GFP-RanBP2Δ6ZFR (B). 48 h post-transfection cells were fixed and immunostained for HA and GFP using specific antibodies. Cells expressing detectable levels of either protein alone or together were tabulated. For each experiment, the expression of at least 400 cells was tabulated. (TIF) [file pbio.1001545.s004.tif]

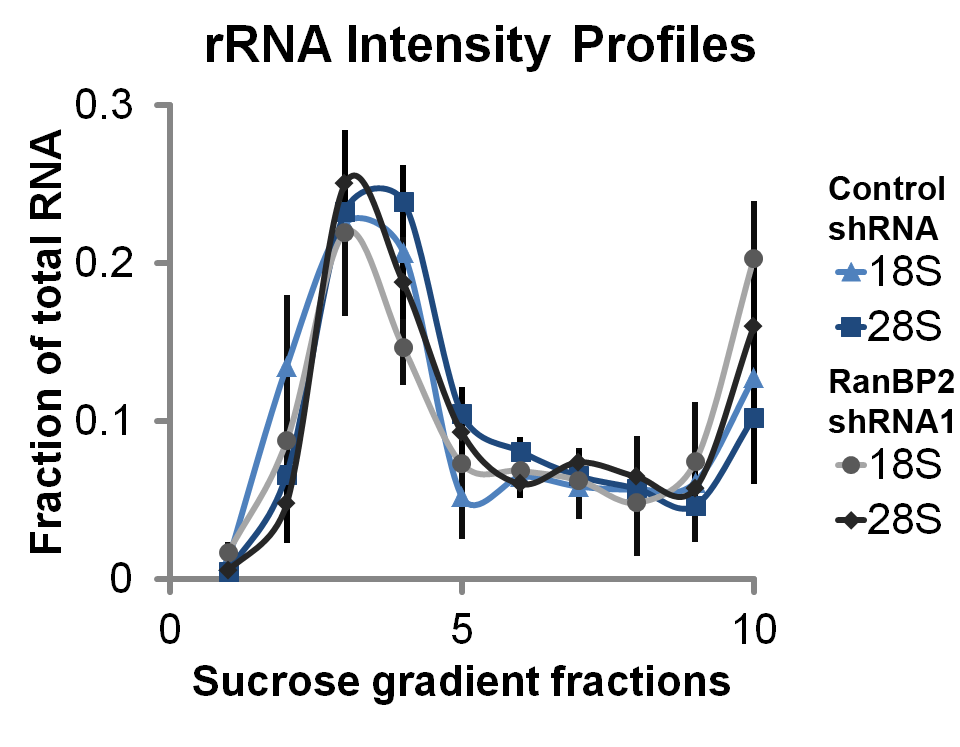

Supplement: Figure S5 — Densitometry analysis of ribosome intensities in sucrose gradients. For each polysome gradient, ribosomal rRNA was visualized as in Figure 7A and 7B and the intensity of the 28S rRNA and 18S rRNA bands were calculated by densitometry analysis. The fraction of the ribosomal rRNAs in each sucrose gradient fraction was then averaged between experiments. Each data point represents the average and standard error between three independent experiments. (TIF) [file pbio.1001545.s005.tif]

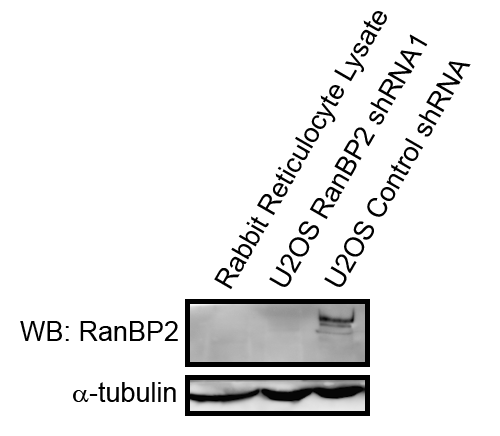

Supplement: Figure S6 — Rabbit reticulocyte lysate does not contain RanBP2/Nup358. Rabbit reticulocyte lysates, and lysates from U2OS cells treated with shRNA1 against RanBP2 or control lentiviruses for 4 d, were separated by SDS-PAGE and immunoblotted for RanBP2 and α-tubulin. (TIF) [file pbio.1001545.s006.tif]
