# Supplementary material for: RanBP2/Nup358 Potentiates the Translation of a Subset of mRNAs Encoding Secretory Proteins
Source: PLoS Biol. 2013 Apr 23;11(4):e1001545. doi: 10.1371/journal.pbio.1001545 (PMC3635865; doi:10.1371/journal.pbio.1001545)
Supplement: Text S1 — Additional supplemental materials and methods. (DOC) [file pbio.1001545.s009.doc]

**Supplemental Materials and Methods**

**DNA plasmids and generation of *ftz* and *CALR* constructs**

For all expression experiments, pCDNA3.1 plasmid containing various versions of the *ftz* gene [1,2], pEGFP plasmid containing the *H1B-GFP* fusion gene [3], or pSPORT6 plasmid containing the *calreticulin (CALR)* gene (OpenBiosystems), were used. To alter the SSCR of the *ftz* and *CALR* genes (see Supplemental Table 1), restriction enzyme free cloning was performed, as described elsewhere [4]. To insert the HA coding sequence into the *CALR* gene, restriction enzyme free cloning was performed using forward and reverse oligonucleotides of the GGCCAAGGACGAGCTGTACCCATACGATGTTCCAGATTACGCTTAGAGAGGCCTGCTCC sequence. For shRNA rescue experiments a plasmid containing GFP-RanBP2 [5], was used. In addition a mutant version lacking zinc fingers 3-8, which lacks 4371-5694 nucleotides of the human RanBP2 ORF, was constructed by digesting the GFP-tagged full length human RanBP2 with SwaI restriction enzyme and ligating the resulting vector.

**Cell culture, transfection and RNA interference by shRNA**

COS7 and U2OS cells were maintained in Dulbeco’s Modified Eagle Medium (DMEM) supplemented with 10% fetal bovine serum. NIH 3T3 cells were maintained in DMEM supplemented with 10% calf serum. Cells were plated overnight on 35-mm-diameter dishes or acid washed 1.5 coverslips and transfected with 1μg of either *ftz* or *CALR-HA* plasmid alone, or with 1μg of *H1B-GFP* plasmid, using GenJet in vitro transfection reagent for U2OS cells (SignaGen Laboratories) following the manufacturer’s protocol. For fluorescence *in situ* hybridization (FISH) and immunofluorescence, cells were incubated 16-24 hours post transfection then washed with phosphate buffered saline (PBS) three times and fixed in 4% paraformaldehyde in PBS at room temperature. For protein and RNA analysis, cells were split into two plates 24 hours post transfection. Then at 24 hours post transfection, cells were washed three times with ice-cold PBS and lysed with Laemmli sample buffer or treated with PureLink RNA Mini Kit (Ambion) using the manufacture’s protocol to isolate total RNA.

For lentiviral delivered shRNA, plasmids encoding shRNA against RanBP2 (shRNA1: TRCN0000003452, shRNA2: TRCN0000003453, shRNA3: TRCN0000003454, Sigma), UAP56 (TRCN0000286276, Sigma), URH49 (TRCN0000333248, Sigma) or empty vector (pLKO.1) were transfected into the HEK293T cells together with the accessory plasmids, VSVG and Δ8.9, to generate lentivirus. Supernatant medium was collected 48hr post-transfection and filtered through a 0.44 µm filter. For infection, lentivirus-containing medium was applied to U2OS cells with 8 µg/ml hexadimethrine bromide. Puromycin was applied 24 hr post-infection at 2 µg/ml to select for infected cells. Poly(A) distribution, *ftz* mRNA export and levels of knockdown proteins were assessed 4 days post-infection. For the evaluation of protein expression, cells were transfected with plasmids containing the *ftz*/*CALR-HA*/*H1B-GFP* genes 3 days post-infection and lysates were collected 4 days post-infection and assessed for protein levels by immunoblot and mRNA levels by northern blot.

**Deglycosylation assay**

U2OS cells were transfected with plasmids containing *MHC-ftz* or a frame-shifted *MHC-ftz* (*FS-MHC-ftz*, see [1] and Supplemental Table 1). This second construct includes a nucleotide addition at the beginning of the SSCR, altering the encoded polypeptide, and a nucleotide deletion at the end of the SSCR, to restore the reading frame for the rest of the *ftz* ORF. 24 hrs post transfection cells were lysed with 1X RIPA lysis buffer (50mM Tris-HCl pH 8.0, 150mM NaCl, 1% NP-40, 1% sodium deoxycholate, 0.1% SDS) and clarified by centrifugation at 10,000 *g*, for 30 min at 4ºC. The lysate comprising 1/6 of a transfected 35 mm dish was treated with either Endoglycosidase H (1000units; New England Biolabs) or Protein N-glycanase F (500 units; New England Biolabs) at 37ºC for two hours. The reacted protein was precipitated with trichloroacetic acid (TCA), washed with acetone and denatured in Laemmli sample buffer. Note that the heterogeneity in FS-MHC-ftz protein levels was likely due to an incomplete recovery of protein after TCA precipitation.

***In vitro* translation**

The *in vitro* translation was performed as described in the manufacturer’s protocol (Promega, Catalogue number L4960). Briefly, the *in vitro* transcription and the purification of capped *MHC-ftz* mRNA and the *2Ile-MHC-ftz* mRNA were performed as mentioned above. Each 50µl radioactive *in vitro* translation reaction mixture contained 35 µl rabbit reticulocyte lysate, 0.02mM amino acid mixture minus cyteine, 5 µl of [^35^S] ]-cysteine (1,200 Ci/mmol) at 10mCi/ml, 40U RNaseout Ribonuclease inhibitor, 2µg of the denatured template mRNA and 4µl of RNase free water. The translation reaction mixture was incubated at 30ºC for 90minutes. A control reaction with no mRNA was used to measure the background incorporation of labeled amino acids. At the end of the incubation, 5 µl of the reaction mixture was mixed with 1 µl of 6XLaemmli sample buffer and denatured at 65ºC for 10minutes and loaded onto a 12%SDS-PAGE. Autoradiograms of the dried gels were developed after exposure of two days.

**Cycloheximide chase assay**

U2OS cells were transfected with *MHC-ftz* or the *2Ile-MHC-ftz* plasmids. Following 20 hours of transfection, the transfected cells were treated with 100 µM cycloheximide and the cell lysates were collected in 1X Laemmli sample buffer at the indicated time points. The protein samples were then denatured, separated on a 12%SDS-PAGE and detected by immunoblot. Protein levels were computed using densitometry anaylsis using ImageJ.

***In vitro* mRNA synthesis, and cell microinjection**

*In vitro* transcription, polyadenylation and purification of capped *MHC-ftz* and *c-ftz* mRNA was performed as previously described [1]. COS7 and NIH 3T3 cells were microinjected as previously described [6], with mRNA (200 ng/μl), 70kDa FITC-dextran (Invitrogen Corp.) in Injection Buffer (10 mM HEPES, pH 7.4, 100mM KCl), with or without pre-spun (15min at 10,000 *g*) 30% HeLa nuclear extract (prepared as previously described [7]). Cells were then incubated at 37°C for various times, then washed three times with PBS, and fixed with 4% paraformaldehyde in PBS for 15 min.

**FISH, immunofluorescence, and imaging**

After fixation, cells were washed three times with PBS and permeabilized with 0.1% TritonX-100 in PBS for 15min. Cells were stained for *ftz* mRNA by FISH, as previously described [1,6] using Alexa546-conjugated probes against ftz (GTCGAGCCTGCCTTTGTCATCGTCGTCCTTGTAGTCACAACAGCCGGGAC AACACCCCAT) or CALR (CAGATGTCGGGACCAAACATGATGTTGTATTCTGAGTC TCCGTGCATGTC). Immunostaining, was performed as previously described [6], using antibodies against HA (HA-7 mouse monoclonal from Sigma, 1:1000), FLAG (M2 monoclonal from Sigma, 1:1000), Trapα (rabbit polyclonal, see [8], 1:1000), TIA-1 (goat polyclonal from Santa Cruz Biotechnology, inc., 1:500), eIF3B (goat polyclonal from Santa Cruz Biotechnology, inc., 1:1000), GFP (rabbit polyclonal, Invitrogen, 1:500) and then probed with Alexa488-, Alexa546- or Alexa647-conjugated secondary antibodies (Invitrogen, 1:500). Imaging, and nuclear mRNA export quantification were performed as previously described [1,6].

**Determination of mRNA partitioning to different subcellular compartments**

To analyze the extent of ER, nuclear and non-ER cytosolic mRNA (Figure 7E and 8E), cells were either fixed in paraformaldehyde in order to determine the total cytoplasmic (C) and nuclear (N) levels of mRNA, or extracted to determine the amount of ER-associated mRNA (ER). For extraction, cells were washed three times with CHO buffer (115 mM KAc, 25 mM HEPES pH 7.4, 2.5 mM MgCl_2_, 2 mM EGTA and 150 mM Sucrose) at 37°C then permeabilized in CHO buffer with 0.025% (w/v) digitonin (Sigma) for 10 seconds on a 40°C heated block, and then fixed in 4% paraformaldehyde in PBS as described previously [9,10]. Cells were FISH stained, imaged, and the levels of cytoplasmic, nuclear and ER-bound mRNA were tabulated as described previously[6,10]. Using the average level of cytoplasmic and ER-associated FISH intensities, the amount of cytoplasmic non-ER mRNA was tabulated (= C - ER). Using the average intensities in the three fractions the average total RNA levels and fraction in each compartment were calculated.

**Immunoblotting**

After 16-24hrs of transfection, cells were lysed in Laemmli sample buffer and separated on a 12% SDS polyacrylamide gel. Protein was transferred to nitrocellulose membrane and probed with primary antibodies against HA (HA-7 mouse monoclonal, Sigma, 1:4000 dilution), GFP (rabbit polyclonal, Invitrogen, 1:1000 dilution), phospho-eIF2α (rabbit polyclonal, Cell Signaling, 1:1000 dilution), αTubulin (DM1A mouse monoclonal, Sigma, 1:250 dilution), mAb414 (mouse monoclonal, Cederlane, 1:5000 dilution), RanBP2 (goat polyclonal, [11], 1:1000 dilution), RanGAP1 (goat polyclonal, [11], 1:500 dilution), Ran (mouse monoclonal, BD Transduction Laboratories, 1:1000 dilution), UAP56 (rat polyclonal, [12], 1:100 dilution), URH49 (rat polyclonal, [12], 1:100 dilution), F1 ATPase synthase subunit α (mouse monoclonal, Sigma, 1:1000), Trapα (rabbit polyclonal, [8], 1:1000), GRP78/BiP (rabbit polyclonal, abcam 1:25) or lamin A/C (goat polyclonal, Santa Cruz, 1:1000) and then horse radish peroxidase conjugated anti-mouse, -rabbit, -rat or -goat secondary antibodies (Cell Signaling). Blots were visualized with chemiluminescence luminol reagent (Pierce) and a Versadoc system (Bio-Rad). Densitometry analysis was performed using ImageJ.

**Northern blotting**

For northern blots, RNA was extracted from a 6 well dish (~4.5 μg) and separated on 1% agarose gel in 1x MOPS buffer (22 mM MOPS, 5 mM sodium acetate, 0.5 M EDTA) with 3% formaldehyde. Samples were transferred to a nitrocellulose membrane using capillary action and 20x SSC (1x SSC: 150 mM NaCl and 15 mM NaCitrate pH 7.1). Blots were then incubated for 2 hrs in Church buffer (0.5 M phosphate buffer pH 7.2, 7% SDS, 1% BSA, and 1 mM EDTA) and then probed overnight at 65ºC in 5ml Church buffer with radiolabeled oligonucleotide probes for *GFP*, *ftz* and *CALR*. To generate the probes for northern blots, PCR products from *GFP*, *ftz* and *CALR* (40-500ng) were converted to radiolabeled oligonucleotide probes using [α^32^P]dATP and the Prime-a-gene labeling system (Promega). Probes generated from each reaction were purified using G-25 MicroSpin Columns (GE Healthcare), then denatured at 95ºC for 3minutes and used immediately in the probing solution. After probing, the membranes were washed three times with northern blot wash buffer (0.4x SSC, 0.1% SDS), and exposed on a phosphoimager cassette that was imaged with a Typhoon phosphoimager system.

**Identification of ALREX-binding proteins**

To prevent non-specific binding of nuclear extract components to the beads, 100 μl HeLa nuclear extract (7 mg/ml), which was prepared as previously described [7,13], was mixed with 10 μl denatured E. Coli tRNA (20 mg/ml, Sigma), 10 μl Salmon Sperm DNA (11 mg/ml, Sigma), 5 μl of RNase-free BSA (20 mg/ml, Ambion), and 250 μl of 2x binding buffer (0.1% TritonX-100, 1.2 M NaCl, 10 mM MgCl_2_, 2 mM DTT, Figure 3E-G) or 250 μl of 2x low-salt binding buffer (0.1% TritonX-100, 0.4 M NaCl, 10 mM MgCl_2_, 2 mM DTT, Supplemental Figure 3). 20 μl of unbound streptavidin-coated magnetic beads (Dynabeads, Invitrogen) were added to pre-clear the nuclear extract. After incubating the solution for 15 min at 4°C with gentle rotation, the beads were removed. The pre-clearance step was repeated 5 times (i.e. each time adding an extra 20 μl of beads) to remove all non-specific binding. The solution was then mixed with 20 μl of beads that were pre-bound with 10 μg of *Ins*, *7A-Ins* and βG biotinylated RNA. The solution was incubated for 1 hour at 4°C with gentle rotation. The beads were then washed five times by incubation with either 500 μl of binding buffer (0.1% TritonX-100, 600mM NaCl, 5mM MgCl_2_, 1 mM DTT; Figure 3E), 500 μl of binding buffer with elevated salt (0.1% TritonX-100, 800mM NaCl, 5mM MgCl_2_, 1 mM DTT; Figure 3F-G), or 500 μl of low-salt binding buffer (0.1% TritonX-100, 200mM NaCl, 5mM MgCl_2_, 1 mM DTT; Supplemental Figure 3). The beads were then isolated, treated with 10 μl of RNase solution (0.1% TritonX-100, 100 mM NaCl, 1 mg/ml RNase A, Sigma) for 15 min at room temperature. The supernatant was removed and mixed with 10 μl of 2x Laemmli sample buffer. The isolated beads were also mixed with 20 μl of 2x Laemmli sample buffer. All samples were denatured at 90°C for 5 min and separated by SDS-PAGE on a 4-20% gradient gel. The gel was either silver stained (SilverQuest, Invitrogen) or transferred to nitrocellulose for immunoblotting. All silver-stained protein bands were cut and identified by microcapillary liquid chromatography tandem mass spectrometry (Taplin Mass Spectrometry Facility, Harvard Medical School).

**Expression and purification of RanBP2 fragments and Ran**

The RanBP2 TPR domain (amino acid residues 1-601), RBR1 (residues 514-1245), ZFD (residues 1335-1829), RBR2 (residues 1832-2553), C-Term (residues 2765-3138) were amplified from the pBSK-RanBP2 [11] and cloned into pET28a vector (Novagen) using restriction-free cloning with the addition of N-terminal His-tag. Each construct was expressed in *E. coli* BL21 cells and cell pellets were lysed by French press in protein purification buffer (1%(v/v) TritonX-100, 50mM HEPES pH8.0, 5mM MgCl_2_, 100mM KCl and 20mM imidazole). The recombinant proteins were eluted in elution buffer (250mM imidazole, 50mM HEPES PH8.0, 5mM MgCl_2_, 100mM KCl) and subsequently dialyzed in storage buffer (50mM HEPES pH 8.0, 5mM MgCl_2_, 100 mM KCl). The GST-E3 RanBP2 fragment (residues 2553-2838) was expressed in BL21 cells and purified as previously described [11]. GST-Ran was expressed in BL21 cells and purified as previously described [14]. To load Ran with nucleotides, the purified recombinant protein was incubated in 100mM NaCl, 50 mM HEPES pH7.5, EDTA to 25mM, DTT to 1mM, and GTP/GDP (100x molar excess over protein). The solution was incubated on ice for 40 min then MgCl_2_ was added to bring the final concentration upto 50mM. The solution was subsequently dialyzed overnight in 100 mM KCl, 50 mM HEPES pH 7.5, and 5 mM MgCl_2_.

**RNA synthesis, EMSA**

For biotinylated RNA, 1 μg of forward and reverse DNA oligonucleotides (Ins: GCTAATACGACTCACTATAGGACCATGGCCCTGTGGATGCGCCTCCTGCCCCTGCTGGCGCTGCTGGCCCTCTGGGGACCTGACCCAGCCGCAGCC, 7A-Ins: GCTAATACGACTCACTATAGGACCATGGCACTGTGGATGCGACTCCTACCCCTACTGGCACTGCTAGCCCTATGGGGACCTGACCCAGCCGCAGCC, βg: GCTAATACGACTCACTATAGGCTTATGGTGCATCTGACTCCTGAGGAGAAGTCTGCCGTTACTGCCCTGTGGGGCAAGGTGAACGTGGATGAAGCC) were denatured at 90°C and re-annealed by slowly cooling down the solution to room temperature. The oligonucleotides were transcribed into RNA using T7 RNA polymerase (Ambion) in the presence of 1 mM ATP, GTP, UTP, 0.25 mM CTP and 4 mM biotin-11-CTP (PerkinElmer) at 37°C for 3 hrs. After treating the samples with DNase, RNA was purified using Illustra MicroSpin G-25 columns (GE Healthcare). For EMSA experiments, the SSCR sequence of insulin (ACCATGGCCCTGTGGATGCGCCTCCTGCCCCTGCTGGCGCTGCTGGCCCTCTGGGGACCTGACCCAGCCGCAGCC) and the insulin 7A mutant (ACCATGGCACTGTGGATGCGACTCCTACCCCTACTGGCACTGCTAGCCCTATGGGGACCTGACCCAGCCGCAGCC), along with the control sequence from β-globin (CTTATGGTGCATCTGACTCCTGAGGAGAAGTCTGCCGTTACTGCCCTGTGGGGCAAGGTGAACGTGGATGAAGCC) were cloned by restriction-free cloning between the *HindIII* and *XhoI* sites of pCDNA3. These plasmids were digested with XhoI and transcribed using RNA using T7 RNA polymerase (Ambion) in the absence or presence of 0.4 μCi/μl [α^32^P]-GTP (all three constructs contained similar levels of G). For the *MHC* RNA, *UUG-ftz* in pCDNA3 [1] digested with NcoI, was used as a template. Synthesized RNA products were denatured, resolved by polyacrylamide gel electrophoresis (TBE, 3.5 or 10% acrylamide; acrylamide/bisacrylamide ratio of 19:1) and then gel isolated. The labeled RNA was incubated with Hela cell nuclear extract (0.44 μg/μl final protein concentration) RanBP2 fragments (50ng/μl in Figure 4C-E, 120ng/μl in Figure 4F) or BSA (120 ng/μl) in 1.5x φ buffer (1x φ buffer: 150 mM KAcetate, 5 mM MgAcetate, 20 mM HEPES pH 7.4), with 10 μg/ml denatured yeast tRNA at room temperature for 15 min. For the competition EMSA experiment, unlabeled RNA was first mixed with tRNA and radiolabeled RNA, then incubated with nuclear extract or recombinant proteins. For the Ran competition assay, 170 ng/μl BSA or GST-Ran was pre-incubated with 50 ng/μl RanBP2 ZFD, then incubated with labeled RNA and tRNA as described previously. EMSA was performed by native polyacrylamide gel electrophoresis (TBE, 3.5%, 5% and 10% acrylamide; acrylamide/bisacrylamide ratio of 19:1). The TBE gels were visualized using a Typhoon phosphorimager (GE Healthcare).

**Cell fractionation and [^35^S]-labeling of newly synthesized proteins**

Control or shRNA-treated U2OS cells (6 cm dish, 60-100% confluent) were incubated with 10 μCi of [^35^S]-methionine/cysteine in methionine/cysteine-free DMEM medium (10% FBS) for 15 min at 37ºC. Then the cells were trypsinized, pelleted (800 *g,* 3 min), washed twice with PBS + 0.1 mg/ml soybean trypsin inhibitor (Sigma) and once with 1x φ buffer. The washed cell pellet was resuspended in 200µl of 1x φ buffer and then mixed with 200µl of 1x φ buffer with 0.2% digitonin. The lysed cells were centrifuged at 800 *g* for 3 min to generate a pellet (nuclei, ER and associated mitochondria) and supernatant (cytoplasm and non ER-associated mitochondria). The supernatant was centrifuged 10,000 g for 3 min to remove mitochondria. The pellet fraction was resuspended in 200µl 1x φ buffer and mixed with an equal volume of 1x φ buffer with 0.5% TritonX-100. The solution was incubated on ice for 5 min to disrupt the ER followed by centrifugation at 800 *g* for 3 min. The resulting pellet contains the nuclear fraction, while the supernatant contains ER and mitochondrial proteins. The total protein concentration in each fraction was quantified by either BCA protein reagent (Novagen) or by separating the proteins by SDS-PAGE followed by Coomassie blue stain. The cytoplasmic fraction was further purified by TCA precipitation. Then the newly synthesized proteins were assessed by measuring [^35^S] levels using a liquid scintillation counter. Fractions were also denatured in Laemmli sample buffer, separated by SDS-PAGE and analyzed by immunoblot.

**Immunopreciptation of endogenous proteins**

U2OS cells were washed with 1XPBS. Then the cells were starved with 2ml DMEM low in Cysteine/methionine for 20 minutes and then labeled with 100-200μCi/ml [^35^S] for 20 additional minutes. Following the incubation, the cells were washed with 1X ice cold PBS and lysed in 500μl buffer containing 20mM pH 7.5 HEPES, 150 mM NaCl, 10mM iodoacetamide, 1X Protease inhibitor (Roche), 1% NP-40 with rocking at 4°C for 20min. The cell debris was removed by centrifugation at 16,000 *g* for 10 min at 4°C. The cleared cell lysate was split into two equal portions. One portion was incubated with 20 μl mouse anti α-tubulin antibody (mouse monoclonal DM1A, Sigma) and the other portion with 15 μl of rabbit anti-BiP antibody (rabbit polyclonal, AbCam). The mixture was incubated at 4°C for 20min. Then 50ul of the protein G (for mouse anti-tubulin) or protein A (rabbit anti-BiP) beads were added in the lysis-antibody mixture respectively and incubated with rocking at 4°C overnight. The next day, the incubated protein beads were washed three times with wash buffer (20mM Hepes NaOH pH7.5, 150mM NaCl). The samples were separated by SDS-PAGE and visualized using a Typhoon phosphorimager (GE Healthcare).

**Polysome analysis**

U2OS cells were cultured at 25-30% confluence in a 100mm dish and were infected with lentivirus containing control or RanBP2 gene specific shRNA1 plasmids. Following 24 hours of infection, the cells that were resistant against 2.0 µg/ml puromycin were selected. On the third day of puromycin treatment, the cells were transfected with plasmids containing *MHC-ftz* constructs together with plasmids containing *histone 1B GFP*. Following 24 hrs of transfection, cells were incubated with 100 µg/ml cycloheximide for 15 minutes at 37ºC then washed three times with ice cold HBSS (Hank’s Balanced Salt Solution, Gibco) with 100 µg/ml cycloheximide in 4 ºC and lysed directly on the plate using 1X polysome lysis buffer (20mM HEPES at pH 7.6, 125mM KCl, 5mM MgCl_2_, 1mM DTT, 0.1mg/ml cycloheximide, 1X protease inhibitor [Roche Protease Inhibitor cocktail tablet], 1% NP-40, 200U of RNaseout [Invitrogen]) and gentle scraping. For each construct, 0.6ml of 1X polysome lysis buffer was added and the lysates were collected in eppendorf tubes and incubated in cold room shaker for 15 min. The nuclei and debris were removed by centrifugation at 10,000 *g* for 10 min. 0.6 ml of the supernatant was layered onto a 10.5 ml sucrose gradient (25-45%) and centrifuged at 200,000 *g* for 2.5 hr. Fractions (1.1ml) were collected manually from the top and treated with 48µl of 20% SDS, 24 µl of 0.5M EDTA pH 8.0 and 7.6 µl of proteinase K (20mg/ml) for 30 min at room temperature. RNA was precipitated by adding 120 µl Sodium acetate (3M pH5.2), 2.5 µl glycogen and 2400 µl of 100% ethanol and incubating the samples overnight at -20ºC. The samples were then centrifuged at 13,000 *g* for 10 min at 4ºC. The RNA pellet was dissolved is RNase free water and analyzed by northern blot.

**Analysis of number of RanBP2 zinc finger domain and length of longest non-A stretch**

The number of RanBP2 zinc finger domains for each of the 38 species was determined by blasting the human RanBP2 sequence against the organisms’ genome in ENSEMBL ([http://www.ensembl.org](http://www.ensembl.org/)). Only species that contained a single unambiguous full length RanBP2 sequence were used. The number of zinc fingers were determined using pfam (<http://pfam.sanger.ac.uk/>) and by visual inspection.

For these 38 species, transcript sequences were downloaded from Ensembl Biomart (<http://www.biomart.org/index.html>) and Saccharomyces Genome Database (SGD for *S. cerevisae*) (<http://www.yeastgenome.org/>). With the exception of *S. cerevisae*, and *A. pisum* all transcript sequence were retrieved from Ensembl Gene 64 through Galaxy (<http://main.g2.bx.psu.edu/>) on Dec 2010. *A. pisum* and *S. cerevisae* transcript sequences were retrieved from Ensembl Metazoa 11 and from SGD, respectively. For each protein-coding region starting with an ATG and with a length greater than 198 nt, we calculated the length of the longest stretch of nucleotides without adenines for the 5’ most 99 nucleotides and a randomly selected downstream 99 nucleotides. Signal sequence coding regions were determined by annotation in Ensembl Biomart (Dec 2010) and SGD (*S. cerevisae*; Jan 2012). For, *D. discoideum* the earlier version of Ensembl dataset had an error in signal peptide annotations such that ~95% of all transcripts were annotated as containing signal peptides. This problem has been recently fixed, therefore for this organism we used signal peptide annotations retrieved from Ensembl Protist 12 on Jan 2012. For each organism, the average and the standard error of the mean of the longest non-adenine tract across all transcripts with signal sequence regions were calculated and given in Table S3.

**References:**

1. Palazzo AF, Springer M, Shibata Y, Lee C-S, Dias AP, et al. (2007) The signal sequence coding region promotes nuclear export of mRNA. PLoS Biol 5: e322. doi:10.1371/journal.pbio.0050322.

2. Cenik C, Chua HN, Zhang H, Tarnawsky S, Akef A, et al. (2011) Genome analysis reveals interplay between 5’UTR introns and nuclear mRNA export for secretory and mitochondrial genes. PLoS Genetics 7: e1001366. doi:10.1371/journal.pgen.1001366.

3. Contreras A, Hale TK, Stenoien DL, Rosen JM, Mancini MA, et al. (2003) The dynamic mobility of histone H1 is regulated by cyclin/CDK phosphorylation. Mol Cell Biol 23: 8626–8636.

4. Van den Ent F, Löwe J (2006) RF cloning: a restriction-free method for inserting target genes into plasmids. J Biochem Biophys Methods 67: 67–74. doi:10.1016/j.jbbm.2005.12.008.

5. Joseph J, Dasso M (2008) The nucleoporin Nup358 associates with and regulates interphase microtubules. FEBS Lett 582: 190–196. doi:10.1016/j.febslet.2007.11.087.

6. Gueroussov S, Tarnawsky SP, Cui XA, Mahadevan K, Palazzo AF (2010) Analysis of mRNA nuclear export kinetics in mammalian cells by microinjection. J Vis Exp 46: 2387. doi:10.3791/2387.

7. Krainer AR, Maniatis T, Ruskin B, Green MR (1984) Normal and mutant human beta-globin pre-mRNAs are faithfully and efficiently spliced in vitro. Cell 36: 993–1005.

8. Görlich D, Prehn S, Hartmann E, Herz J, Otto A, et al. (1990) The signal sequence receptor has a second subunit and is part of a translocation complex in the endoplasmic reticulum as probed by bifunctional reagents. J Cell Biol 111: 2283–2294.

9. Cui XA, Zhang H, Palazzo AF (2012) p180 Promotes the Ribosome-Independent Localization of a Subset of mRNA to the Endoplasmic Reticulum. PLoS Biol 10: e1001336. doi:10.1371/journal.pbio.1001336.

10. Cui XA, Palazzo AF (2012) Visualization of Endoplasmic Reticulum Localized mRNAs in Mammalian Cells. J Vis Exp 70: e50066. doi:10.3791/50066.

11. Pichler A, Gast A, Seeler JS, Dejean A, Melchior F (2002) The nucleoporin RanBP2 has SUMO1 E3 ligase activity. Cell 108: 109–120.

12. Yamazaki T, Fujiwara N, Yukinaga H, Ebisuya M, Shiki T, et al. (2010) The closely related RNA helicases, UAP56 and URH49, preferentially form distinct mRNA export machineries and coordinately regulate mitotic progression. Mol Biol Cell 21: 2953–2965. doi:10.1091/mbc.E09-10-0913.

13. Mayeda A, Krainer AR (1999) Preparation of HeLa cell nuclear and cytosolic S100 extracts for in vitro splicing. Methods Mol Biol 118: 309–314. doi:10.1385/1-59259-676-2:309.

14. Wilde A, Zheng Y (1999) Stimulation of microtubule aster formation and spindle assembly by the small GTPase Ran. Science 284: 1359–1362.
